# An exact solution for *R*_2,_*_eff_* in CPMG experiments in the case of two site chemical exchange

**DOI:** 10.1016/j.jmr.2014.02.023

**Published:** 2014-07

**Authors:** Andrew J. Baldwin

**Affiliations:** Physical and Theoretical Chemistry Laboratory, University of Oxford, Oxford OX1 3QZ, UK

**Keywords:** Chemical exchange, CPMG experiments, Carver Richards equation, Two-site exchange

## Abstract

The Carr–Purcell–Meiboom–Gill (CPMG) experiment is widely used to quantitatively analyse the effects of chemical exchange on NMR spectra. In a CPMG experiment, the effective transverse relaxation rate, *R*_2,_*_eff_*, is typically measured as a function of the pulse frequency, *ν_CPMG_*. Here, an exact expression for how *R*_2,_*_eff_* varies with *ν_CPMG_* is derived for the commonly encountered scenario of two-site chemical exchange of in-phase magnetisation. This result, summarised in Appendix A, generalises a frequently used equation derived by Carver and Richards, published in 1972. The expression enables more rapid analysis of CPMG data by both speeding up calculation of *R*_2,_*_eff_* over numerical methods by a factor of ca. 130, and yields exact derivatives for use in data analysis. Moreover, the derivation provides insight into the physical principles behind the experiment.

## Highlights

• An exact equation for two site exchange for use in analysing CPMG data.
• A generalisation of a result derived by Carver and Richards in 1972.
• Offers physical insights into how CPMG experiment work.
• Provides a means for significantly more rapid analysis of experimental CPMG data.

## Graphical abstract

**Figure:**
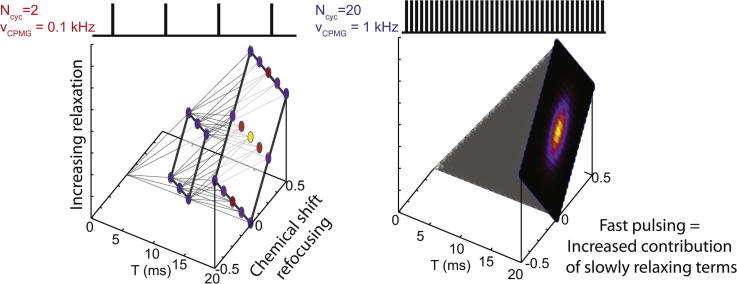

## 1 Introduction

Many chemical systems analysed by NMR spectroscopy spontaneously undergo dynamical changes that lead to variation in the isotropic chemical shift over time. When the frequency of these processes is similar to the frequency of the chemical shift difference, interference effects lead to changes in the intensity, linewidth and frequency of observed resonances. Collectively termed chemical exchange phenomena, these effects can be quantitatively probed with suitable experiments to provide insight into the underlying molecular processes [1,2]. CPMG experiments [3,4] are a notable example [5] that can provide kinetic and thermodynamic information describing the exchange process, and also structures of the interconverting states [29–32], even when the population of one of the interconverting conformers is as low as 1%.

As anticipated in an early piece of theoretical work [6], CPMG experiments have led to important insights into biomolecular processes. These methods have revealed sparsely populated conformational states, termed ‘excited’ states, in proteins have been identified that are critical for functions as diverse as enzymatic catalysis [7–9], molecular recognition [10], quaternary dynamics [11–13] and protein folding [14–17]. Extensive efforts over recent years has resulted in a number of individually tailored CPMG experiments and associated labelling schemes to measure not only isotropic chemical shifts of excited states [18–24] but also structural features such as bond vector orientations [25–28]. These experiments together enable elucidation of structures of these hitherto unknown, but functionally important biomolecular conformational states [29–32].

**Fig. 1:**
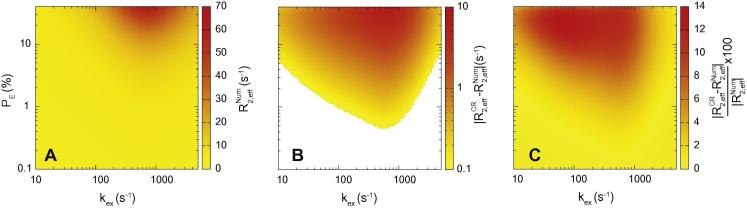
The effective relaxation rate *R*_2,_*_eff_* calculated numerically $\left(R_{2\text{,}\mathrm{eff}}^{\mathrm{Num}}\right)$ and using the Carver Richards equation ($R_{2\text{,}\mathrm{eff}}^{\mathrm{CR}}$, Eq. (49)) in the case where *R*_2_*^G^* = *R*_2_*^E^* = 10 s^−1^, Δ*ω* = 0.5 ppm, with a Lamor frequency of 200 MHz for a range of exchange rates and populations, for *N_cyc_* = 2, *T_relax_* = 20 ms, and so *ν_CPMG_* = 100 Hz. (A) The numerically derived relaxation rate, $R_{2\text{,}\mathrm{eff}}^{\mathrm{Num}}$. (B) The difference in relaxation rates, $\left|R_{2\text{,}\mathrm{eff}}^{\mathrm{CR}}-R_{2\text{,}\mathrm{eff}}^{\mathrm{Num}}\right|$. Under many conditions, notably low *P_E_*, the Carver Richards equation is in excellent quantitative agreement with the data. Outside of this regime, there are significant deviations. The experimental error on *R*_2,_*_eff_* measurements is expected to be on the order of 0.3 s^−1^. The region shown in orange and red correspond to a region where the error is greater than 0.3 s^−1^, indicating where systematic errors in fitted parameters will be incurred. Extensive numerical tests of our new result (Eq. (50)) reveal a maximum error of less than 10^−9^ s^−1^ corresponding to the threshold of computational precision. (C) The percentage error incurred using the Carver Richards equation. (For interpretation of the references to color in this figure legend, the reader is referred to the web version of this article.)

In order to accurately extract meaningful parameters, CPMG data must be related to an appropriate theory. There are two commonly applied approaches to simulate the experimental data. The first relies on closed form solutions to the Bloch–McConnell equations [33] such as the Carver Richards equation [6] (Fig. 1), a result found implemented in freely available software [34–36]. When the population of the minor state exceeds approximately 1% however, calculation errors that are significantly larger than the experimental uncertainty can accumulate when this result is used (Fig. 1), which can lead to errors in the extracted parameters. Further insight has come from results that have been derived in specific kinetic regimes [37,38,42], revealing which mechanistic parameters can be reliably extracted from data in these limits. In addition more recently, an algorithm that constitutes an exact solution has been described [37] derived *in silico* using the analysis software maple. As described in Supplementary Section 8, while exact, this algorithm can lead to errors when evaluated at double floating point precision, as used by software such as MATLAB. While the closed form results described above are relatively fast from a computational perspective, they are approximate. A second approach for data analysis involves numerically solving the Bloch–McConnell equations [15,28], where additional and relevant physics such as the non-ideal nature of pulses [39,40], scalar coupling and differential relaxation of different types of magnetisation are readily incorporated. While the effects of these additional physics can be negligible, their explicit inclusion is recommended, when accurate parameters are required for structure calculations [29–32]. Nevertheless, closed form solutions can provide greater insight into the physical principles behind experiments than numerical simulation. Motivated by this principle, here, an exact solution for the effective transverse relaxation rate in a CPMG experiment, *R*_2,_*_eff_*, in the commonly encountered scenario of two-site exchange of in-phase magnetisation (Eq. (50)) is derived. The result is expressed as a linear correction to the Carver Richards equation (summarised in Appendix A), and algorithms based on this have advantages in both precision and speed over existing formulaic approaches (Supplementary Section 8).

In a CPMG experiment, transverse magnetisation is first created, and then allowed to evolve through a series of spin echoes. In this work it is defined that each consists of two delays of duration of *τ_cp_*, separated by a 180° pulse. A single CPMG element is two concatenated echoes, which in the absence of relaxation and chemical exchange, returns transverse magnetisation to an identical state to which it started. In the complete experiment, *N_cyc_* CPMG elements are further concatenated, leading to a pulsing frequency, *v_CPMG_* = *N_cyc_*/*T_rel_* and the total time of the CPMG element is *T_rel_* = 4*τ_cp_N_cyc_*. The change in signal intensity and hence *R*_2,_*_eff_* due to the exchange process is then monitored as a function of *v_CPMG_*.

In the case of two-site chemical exchange, in the absence of pulses, in-phase magnetisation will evolve at two distinct frequencies. As a useful book keeping exercise, one frequency can be associated with an ensemble of molecules that are primarily (but not entirely) in the majorly populated (ground) state, and the second with an ensemble of molecules that are primarily (but not entirely) in the minorly populated (excited) state. Both ensembles are mixed states whose exact ground/excited ‘composition’ depends explicitly on the exchange parameters. It is shown here that a 180° pulse does not simply invert the chemical shift, as it would a pure state. Instead, it further mixes these two ensembles. Consequently, after the second evolution period, four frequencies emerge from a spin echo, corresponding to magnetisation that started and finished on either the ground or excited states, and that which started on the ground and finished in the excited, or vice versa. While the first two pathways are entirely refocused in terms of their chemical shift, the second two are not. The 180° pulse can therefore be considered ‘leaky’, as not all magnetisation is refocused. When multiple Hahn echoes are concatenated in a CPMG experiment, the number of discrete frequencies increases. The derivation of the CPMG signal intensity relies on determining how ‘leaky’ a single CPMG element is, identifying which frequencies are present at the end, evaluating their weighting factors and calculating how these depend on the details of the exchange process.

Each of the discrete frequencies that emerge from a CPMG block can be associated with a mixture of ground and excited state ensembles. A higher proportion of time spend in the excited state leads to more efficient relaxation, and loss of signal intensity. As a consequence, only frequencies that arise predominantly from the ground state ensemble contribute significantly to the observed resonance. At low pulsing frequency, there are few such frequencies. At high pulsing frequency, there are many more such slowly relaxing terms present. It is these slowly relaxing terms that give rise to the characteristic increase in signal observed in a CPMG experiment.

## 2 Derivation

An expression for the effective transverse relaxation rate of the ground state ensemble is sought:

(1) $$R_{2\text{,}\mathrm{eff}}=-\frac{1}{T_{\mathrm{rel}}}\ln\frac{I_{G}(T_{\mathrm{rel}})}{I_{G}(0)}$$

where *T_rel_* is the total time of the concatenated CPMG elements and *I_G_* specifies the signal intensity from the observed ground state at the specified times. In order to calculate the relevant signal intensities a kinetic model for the exchange process and types of magnetisation present need to be specified. The simplest and most widely encountered kinetic scheme is the two-site case for in-phase magnetisation. Here, a ground state and an excited state undergo the conformational rearrangement $G\overset{k_{\mathrm{GE}}}{\underset{k_{\mathrm{EG}}}{⇄}}E$. In this scheme, the exchange rate *k_EX_* = *k_EG_* + *k_GE_* and the fractional populations of the excited (*P_E_*) and ground (*P_G_*) states are given by *k_GE_*/*k_EX_* and *k_EG_*/*k_EX_* respectively. The CPMG experiment consists of a number of free precession elements interspersed with 180° pulses. To evaluate their combined effect, how magnetisation evolves in the absence of pulses needs first to be calculated. This is accomplished most conveniently using the shift basis (*I*^+^ = *I_x_* + *iI_y_* and *I^−^* = *I_x_* − *iI_y_*) using a modified Bloch–McConnell equation [33]:

(2) $$\frac{d}{\mathrm{dt}}\left(\begin{array}{c} I_{G}^{+}\\ I_{E}^{+} \end{array}\right)=R^{+}\left(\begin{array}{c} I_{G}^{+}\\ I_{E}^{+} \end{array}\right)$$

where *E* and *G* denote the magnetisation on the excited and ground states, respectively. The evolution matrix is:

(3) $$R^{+}=\left(\begin{array}{cc} -k_{\mathrm{GE}}-R_{2}^{G} & k_{\mathrm{EG}}\\ k_{\mathrm{GE}} & -k_{\mathrm{EG}}-R_{2}^{E}-i\Delta \omega \end{array}\right)$$

*R*_2_*^G^* and *R*_2_*^E^* specify the intrinsic relaxation of the ground and excited states respectively, and Δ*ω* is the chemical shift difference between the ground and excited states in rad s^−1^. The solution for Eq. (2) is:

(4) $$I(t)=e^{R^{+}t}I(0)=\mathrm{OI}(0)$$

where *I*(0) are *I*(*t*) specify the magnetisation on the ground and excited states at time zero and *t* respectively. Initially the system is in equilibrium, and so $I{\left(0\right)}^{†}=(P_{G}\text{,}P_{E})$ where † indicates a transpose. The derivation of *I*(*t*) first requires the well known matrix *O* (Eq. (17)) that determines how magnetisation evolves during free precession [2]. In the shift basis, the effect of a 180° on-resonance ideal pulse switches magnetisation on *I*^+^ terms to *I^−^*, leading magnetisation to evolve according to the complex conjugate of *R*^+^ (Eq. (3)), (R^+^)^*^. Following a 180° pulse therefore, magnetisation will evolve according to the matrix O^*^. By applying Eq. (4) iteratively, taking the complex conjugate where appropriate, an expression that represents the entire CPMG experiment can be built. This, when used with Eq. (1) enables us to derive an expression for *R*_2,_*_eff_*.

The matrix *M* that represents the CPMG experiment will enable us to evaluate *I*(*t*) = *MI*(0). This can be decomposed into *N_cyc_* concatenated CPMG units, each of which is described by an evolution matrix *P*, such that *M* = *P^Ncyc^*. *M* can be calculated from *P* by diagonalisation to obtain *P_D_*, and then transforming it with its matrix of Eigenvectors *A*, according to:

(5) $$M=P^{N_{\mathrm{cyc}}}={\left({\mathrm{AP}}_{D}A^{-1}\right)}^{N_{\mathrm{cyc}}}={\mathrm{AP}}_{D}^{N_{\mathrm{cyc}}}A^{-1}$$

The CPMG element *P* consists of two concatenated Hahn echoes, *H*, each of which consists of two equal delays of duration *τ_cp_*, separated by a 180° pulse (Eq. (30)):

(6) $$H=O^{*}O$$

The effect of a single CPMG unit is then given by

(7) $$P=H^{*}H={\mathrm{OO}}^{*}O^{*}O$$

as derived in Eq. (42), from which *M* can be calculated using Eq. (5) (Eq. (46)). As implicitly assumed by Carver and Richards, the effects of chemical exchange during signal detection will be neglected (though this assumption can be removed– see Supplementary Section 7), and *I_G_*(*T_rel_*) calculated from:

(8) $$I_{G}(T_{\mathrm{rel}})=M(0\text{,}0)P_{G}+M(0\text{,}1)P_{E}$$

where 0, 0 and 0, 1 specify the required matrix elements of *M*. Insertion of this result into Eq. (1) gives the final result for *R*_2,_*_eff_* (Eq. (50)), summarised in Appendix A. Combining the matrix Eq. (46) with the results in Supplementary Section 7 to give *R*_2,_*_eff_* including the effects of chemical exchange during detection will further improve the theoretical description of the experiment [41].

### 2.1 Determination of O

The free precession matrix *R*^+^ can be related to its diagonalised form *R_D_* via the transformation *R* = *JR_D_J^−^*^1^ such that:

(9) $$O=e^{R^{+}t}=e^{{\mathrm{JR}}_{D}J^{-1}}={\mathrm{Je}}^{R_{D}^{+}t}J^{-1}$$

From which it follows that the matrix exponential is given in terms of two characteristic frequencies, the Eigenvalues *f*_00_ and *f*_11_, corresponding to the ground and excited state ensembles respectively:

(10) $$e^{R_{D}^{+}t}=e^{-{\mathrm{tR}}_{2}^{G}}\left(\begin{array}{cc} e^{-{\mathrm{tf}}_{00}} & 0\\ 0 & e^{-{\mathrm{tf}}_{11}} \end{array}\right)$$

A factor of *R*_2_*^G^* has been factored from both *f*_00_ and *f*_11_, which allows us to express them conveniently in terms of the difference in relaxation, Δ*R*_2_ = *R*_2_*^E^* − *R*_2_*^G^* in what follows and so:

(11) $$\begin{array}{c} f_{00}=\frac{1}{2}(\Delta R_{2}+k_{\mathrm{EX}}+i\Delta \omega )-\frac{1}{2}\sqrt{h_{2}+{\mathrm{ih}}_{1}}\\ f_{11}=\frac{1}{2}(\Delta R_{2}+k_{\mathrm{EX}}+i\Delta \omega )+\frac{1}{2}\sqrt{h_{2}+{\mathrm{ih}}_{1}} \end{array}$$

where

$$h_{1}=2\Delta \omega (\Delta R_{2}+k_{\mathrm{EG}}-k_{\mathrm{GE}})$$

(12) $$h_{2}={\left(\Delta R_{2}+k_{\mathrm{EG}}-k_{\mathrm{GE}}\right)}^{2}+4k_{\mathrm{EG}}k_{\mathrm{GE}}-\Delta \omega^{2}$$

The identity $\sqrt{h_{2}+{\mathrm{ih}}_{1}}=h_{3}+{\mathrm{ih}}_{4}$, enables us to explicitly separate the real and the imaginary components of the Eigenvalues:

$$h_{3}=\frac{1}{\sqrt{2}}\sqrt{h_{2}+\sqrt{h_{1}^{2}+h_{2}^{2}}}$$

(13) $$h_{4}=\frac{1}{\sqrt{2}}\sqrt{-h_{2}+\sqrt{h_{1}^{2}+h_{2}^{2}}}$$

In terms of these substitutions, *f*_00_ and *f*_11_ are then succinctly expressed as:

(14) $$\begin{array}{c} f_{00}=\frac{1}{2}(\Delta R_{2}+k_{\mathrm{EX}}-h_{3})+\frac{i}{2}(\Delta \omega -h_{4})\\ f_{11}=\frac{1}{2}(\Delta R_{2}+k_{\mathrm{EX}}+h_{3})+\frac{i}{2}(\Delta \omega +h_{4}) \end{array}$$

The real part of the two Eigenvalues, *f*_00_*^R^* and *f*_11_*^R^* describe the effective relaxation rates of the two ensembles, and the imaginary parts *f*_00_*^I^* and *f*_11_*^I^* define the frequencies where the resonance will ultimately be observed. The imaginary component, *f*_00_*^I^* denotes the exchange-induced shift in the observed position of the ground state resonance [24]. The following useful sum and difference relations:

(15) $$\begin{array}{cc} & f_{11}^{R}+f_{00}^{R}=\Delta R_{2}+k_{\mathrm{EX}}\\ & f_{11}^{I}+f_{00}^{I}=\Delta \omega \\ & f_{11}^{R}-f_{00}^{R}=h_{3}\\ & f_{11}^{I}-f_{00}^{I}=h_{4} \end{array}$$

play an important role in the CPMG experiment and emerge explicitly as arguments of trigonometric terms in the final expression for *R*_2,_*_eff_* (Eq. (41)). As summarised in Supplementary Section 1, the two frequencies reduce to simple and well-known expressions in the fast and slow exchange regimes, though care must be taken when defining the regime when Δ*R*_2_ ≠ 0. To express the final form of the propagator, two further factors related to the frequencies *f*_00_ and *f*_11_ are defined:

(16) $$\begin{array}{cc} & O_{G}=k_{\mathrm{GE}}-f_{00}\\ & O_{E}=f_{11}-k_{\mathrm{GE}}\\ & N=O_{G}+O_{E} \end{array}$$

and so $O_{G}O_{E}=O_{G}^{*}O_{E}^{*}=k_{\mathrm{EG}}k_{\mathrm{GE}}$, and $N=h_{3}+{\mathrm{ih}}_{4}=\sqrt{h_{2}+{\mathrm{ih}}_{1}}$, a quantity equal to *k_EX_* in the fast exchange limit (Supplementary Section 1). In terms of these variables, the free precession evolution matrix is:

(17) $$O=\frac{e^{-{\mathrm{tR}}_{2}^{G}}}{N}\left(B_{00}e^{-{\mathrm{tf}}_{00}}+B_{11}e^{-{\mathrm{tf}}_{11}}\right)$$

where

(18) $$B_{00}=\left(\begin{array}{cc} O_{E} & k_{\mathrm{EG}}\\ k_{\mathrm{GE}} & O_{G} \end{array}\right)\;\text{and}\;B_{11}=\left(\begin{array}{cc} O_{G} & -k_{\mathrm{EG}}\\ -k_{\mathrm{GE}} & O_{E} \end{array}\right)\text{.}$$

As *O_E_O_G_* = *k_EG_k_GE_*, both *B*_00_/*N* and *B*_11_/*N*are idempotent such that (*B_xx_*/*N*)*^n^* = *B_xx_*/*N* where *xx* = 00, 11. The form of these matrices allows us to gain physical insight into the coefficients. *O_E_*/*N* can be interpreted as a coefficient associated with the proportion of the ensemble that ‘stay’ either in the ground or excited state, within the ensemble, for the duration of the free precession, and *O_G_*/*N* is the coefficient associated with the molecules that effectively ‘swap’ from the ground state ensemble to the excited state, and vice versa, during free precession. Together, these matrices define the ‘composition’ of the mixed ground and excited state ensembles. Both *B*_00_/*N* and *B*_11_/*N* are idempotent and orthogonal, and so when the matrices are raised to a power:

(19) $$O^{n}=\frac{e^{-{\mathrm{ntR}}_{2g}}}{N}\left(B_{00}e^{-{\mathrm{ntf}}_{00}}+B_{11}e^{-{\mathrm{ntf}}_{11}}\right)$$

The observed ground state signal is therefore given by (Eq. (8)):

(20) $$I_{G}(t)=\frac{e^{-{\mathrm{tR}}_{2}^{G}}}{N}\left(e^{-{\mathrm{tf}}_{00}}\left(p_{G}f_{11}+p_{E}(k_{\mathrm{EX}}-f_{00})\right)+e^{-{\mathrm{tf}}_{11}}\left(-p_{G}f_{00}+p_{E}(f_{11}-k_{\mathrm{EX}})\right)\right)$$

**Fig. 2:**
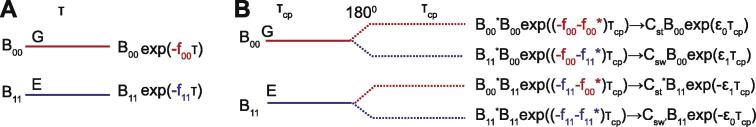
A solid line indicates evolution according to *R*^+^, and a dashed line indicates evolution after a 180° pulse, according (*R*^+^)^*^. (A) During a free precession period in the presence of chemical exchange, signal can be separated into two distinct groups with distinguishable frequencies (Eq. (17)). (B) A Hahn echo is ‘leaky’ in the presence of chemical exchange, in the sense that not all magnetisation is refocused, leading to additional mixing between the ground and excited state ensembles. At the end of the spin echo (Eq. (30)), signal can be separated into four distinguishable frequencies that are defined by differences between either the real (*ε*_0_) or imaginary (*ε*_1_) parts of *f*_00_ and *f*_11_ (Eq. (22)). Magnetisation that ‘stays’ in either the ground or excited state ensemble is refocused (*±ε*_0_), having no imaginary component. However, a proportion of the molecules effectively ‘swaps’ into the other ensemble, and are not complete refocused (*±ε*_1_). The final terms with their weighting coefficients are illustrated. Note that a factor of *exp*((*f*_00_*^R^* + *f*_00_*^I^*)*τ_cp_*) has been factorised from the expression after the arrow.

The spectrum will be a weighted sum of precisely two resonances that evolve with complex frequencies *f*_00_ and *f*_11_ (Fig. 2A). When considering chemical exchange from a microscopic perspective, it is intuitive that any single molecule will not spend all of its time in any one of the two states. Nevertheless, two ensembles can be identified, loosely described as those that spend most of their time on the ground state and those that spend most of their time on the excited state, associated with frequencies *f*_00_ and *f*_11_, and weighting matrices *B*_00_ and *B*_11_, respectively. Armed with *O* (Eq. (19)), expressions for both for a Hahn Echo, and the CPMG propagator can be derived.

### 2.2 Derivation of the spin echo propagator

The basic repeating unit of the CPMG experiment is a Hahn echo, where two delays of duration *τ_cp_* are separated by a 180° pulse, *H* = O^*^O. Two of these are required to give us the CPMG propagator, *P* = H^*^H. *H* can be determined from Eq. (19):

(21) $$H=\frac{e^{-2\tau_{\mathrm{cp}}R_{2}^{G}}}{{\mathrm{NN}}^{*}}\left(B_{00}^{*}e^{-\tau_{\mathrm{cp}}f_{00}^{*}}+B_{11}^{*}e^{-\tau_{\mathrm{cp}}f_{11}^{*}}\right)\left(B_{00}e^{-\tau_{\mathrm{cp}}f_{00}}+B_{11}e^{-\tau_{\mathrm{cp}}f_{11}}\right)$$

Expanding this reveals four discrete frequencies that correspond to sums and differences of *f*_00_ and *f*_11_ (Fig. 2B). That which ‘stays’ in the same ensemble (*exp*(−*τ_cp_*(*f*_00_ + *f*_00_^*^)) or *exp*(−*τ_cp_*(*f*_11_ + *f*_11_^*^))) for the duration will be refocused. That which start in one, then effectively ‘swaps’ after the first 180° pulse will accrue net phase (*exp*(−*τ_cp_*(*f*_00_ + *f*_11_^*^)) or *exp*(−*τ_cp_*(*f*_11_ + *f*_00_^*^))). Note that this terminology should not imply that radiofrequency pulses are affecting the change. It is instead an accounting perspective for describing how the magnetisation will appear. Defining two frequencies, one real and one imaginary:

$${∊}_{0}=-\left(f_{00}^{R}-f_{11}^{R}\right)=h_{3}$$

(22) $${∊}_{1}=-i\left(f_{00}^{I}-f_{11}^{I}\right)={\mathrm{ih}}_{4}$$

then:

(23) $$H=\frac{e^{-\tau_{\mathrm{cp}}\left(R_{2}^{G}+R_{2}^{E}+k_{\mathrm{ex}}\right)}}{{\mathrm{NN}}^{*}}\left((B_{00}^{*}e^{\tau_{\mathrm{cp}}{∊}_{0}}+B_{11}^{*}e^{\tau_{\mathrm{cp}}{∊}_{1}})B_{00}+(B_{11}^{*}e^{-\tau_{\mathrm{cp}}{∊}_{0}}+B_{00}^{*}e^{-\tau_{\mathrm{cp}}{∊}_{1}})B_{11}\right)$$

where the average relaxation rate *exp*(−*τ_cp_*(*f*_00_*^R^* + *f*_11_*^R^*)) = *exp*(−*τ_cp_*(Δ*R*_2_ + *k_ex_*)) has been factored out. At the end of this period, magnetisation that has been entirely refocused will evolve with a purely real frequency, ±*ε*_0_, and magnetisation that has not, will evolve with frequencies ±*ε*_1_. By a similar procedure, the propagator for the second half of the CPMG block can be derived by noting that the complex conjugate of *ε*_1_ is obtained by multiplying it by −1:

(24) $$H^{*}=\frac{e^{-\tau_{\mathrm{cp}}(R_{2}^{G}+R_{2}^{E}+k_{\mathrm{ex}})}}{{\mathrm{NN}}^{*}}\left((B_{00}e^{\tau_{\mathrm{cp}}{∊}_{0}}\text{+}B_{11}e^{-\tau_{\mathrm{cp}}{∊}_{1}})B_{00}^{*}+(B_{11}e^{-\tau_{\mathrm{cp}}{∊}_{0}}+B_{00}e^{\tau_{\mathrm{cp}}{∊}_{1}})B_{11}^{*}\right)$$

Further progress can be made by identifying additional simplifying relations. The elements of idempotent *B*_00_ and *B*_11_ satisfy the condition *B*(1, 0)*B*(0, 1) = *B*(1, 1)*B*(0, 0) where the brackets indicate specific rows and columns of the matrix. In such a case, for a matrix product *AB*, *A* can be replaced by a diagonal matrix *C* such that *AB* = *CB*. As derived in Supplementary Section 2, the two diagonal coefficients of *C* are given by Eq. (66). Dealing with matrix products is cumbersome, and so replacing one of the two matrices with one that is diagonal will be shown to be greatly simplifying (see Eq. (35)). In doing so, the following identities are obtained:

(25) $$\begin{array}{cc} & C_{\mathrm{st}}\cdot B_{00}=B_{00}^{*}\cdot B_{00}\\ & C_{\mathrm{st}}^{*}\cdot B_{11}=B_{11}^{*}\cdot B_{11}\\ & C_{\mathrm{sw}}\cdot B_{00}=B_{11}^{*}\cdot B_{00}\\ & C_{{\mathrm{sw}}^{\prime }}\cdot B_{11}=B_{00}^{*}\cdot B_{11} \end{array}$$

which follow from the definition of ‘stay’ and ‘swap’ diagonal matrices using Eq. (66):

(26) $$C_{\mathrm{st}}=\left(\begin{array}{cc} P_{\mathrm{st}} & 0\\ 0 & P_{\mathrm{st}}^{*} \end{array}\right)\text{,}\;C_{\mathrm{sw}}=\left(\begin{array}{cc} P_{\mathrm{sw}} & 0\\ 0 & P_{{\mathrm{sw}}^{\prime }} \end{array}\right)\text{,}\;C_{{\mathrm{sw}}^{\prime }}=\left(\begin{array}{cc} P_{{\mathrm{sw}}^{\prime }} & 0\\ 0 & P_{\mathrm{sw}} \end{array}\right)$$

The individual matrix elements are given by:

(27) $$\begin{array}{cc} & P_{\mathrm{st}}=O_{G}+O_{E}^{*}=h_{3}-i\Delta \omega \\ & P_{\mathrm{sw}}=O_{G}^{*}-O_{G}=-i(h_{4}-\Delta \omega )\\ & P_{{\mathrm{sw}}^{\prime }}=O_{E}^{*}-O_{E}=-i(h_{4}+\Delta \omega ) \end{array}$$

From these definitions, the following useful identities emerge:

(28) $$\begin{array}{cc} & P_{\mathrm{st}}^{*}O_{G}=P_{\mathrm{st}}O_{G}^{*}\\ & P_{\mathrm{st}}O_{E}=P_{\mathrm{st}}^{*}O_{G}^{*}\\ & P_{\mathrm{sw}}O_{G}^{*}=-P_{{\mathrm{sw}}^{\prime }}O_{E}\\ & P_{{\mathrm{sw}}^{\prime }}O_{G}=-P_{\mathrm{sw}}O_{E}^{*} \end{array}$$

These definitions reveal an important physical interpretation of these cofactors. In the case where magnetisation stays in either the ground or excited state following a 180° pulse, it is multiplied by a ‘stay’ matrix of the form *C_st_*. In the case where magnetisation effectively swaps to the other state, it is multiplied by a ‘swap’ matrix, *C_sw_* or *C_sw_*_′_. The conjugate of either of the swap matrices is obtained by multiplication by −1, leading to the conjugates of Eq. (25):

(29) $$\begin{array}{cc} & C_{\mathrm{st}}^{*}\cdot B_{00}^{*}=B_{00}\cdot B_{00}^{*}\\ & C_{\mathrm{st}}\cdot B_{11}^{*}=B_{11}\cdot B_{11}^{*}\\ & -C_{\mathrm{sw}}\cdot B_{00}^{*}=B_{11}\cdot B_{00}^{*}\\ & -C_{{\mathrm{sw}}^{\prime }}\cdot B_{11}^{*}=B_{00}\cdot B_{11}^{*} \end{array}$$

These operations enable us to arrive at a simplified expression for the two Hahn echo propagators.

(30) $$H=\frac{e^{-\tau_{\mathrm{cp}}\left(R_{2}^{G}+R_{2}^{E}+k_{\mathrm{ex}}\right)}}{{\mathrm{NN}}^{*}}((C_{\mathrm{st}}e^{\tau_{\mathrm{cp}}{∊}_{0}}+C_{\mathrm{sw}}e^{\tau_{\mathrm{cp}}{∊}_{1}})B_{00}+(C_{\mathrm{st}}^{*}e^{-\tau_{\mathrm{cp}}{∊}_{0}}+C_{{\mathrm{sw}}^{\prime }}e^{-\tau_{\mathrm{cp}}{∊}_{1}})B_{11})$$

(31) $$H^{*}=\frac{e^{-\tau_{\mathrm{cp}}(R_{2}^{G}+R_{2}^{E}+k_{\mathrm{ex}})}}{{\mathrm{NN}}^{*}}((C_{\mathrm{st}}^{*}e^{\tau_{\mathrm{cp}}{∊}_{0}}-C_{\mathrm{sw}}e^{{-\tau }_{\mathrm{cp}}{∊}_{1}})B_{00}^{*}+(C_{\mathrm{st}}e^{-\tau_{\mathrm{cp}}{∊}_{0}}-C_{{\mathrm{sw}}^{\prime }}e^{\tau_{\mathrm{cp}}{∊}_{1}})B_{11}^{*})$$

The Hahn echoes in the case of exchange are therefore ‘leaky’ in the sense that they do not completely refocus all the magnetisation. A proportion of the magnetisation ‘stays’ in either the ground or excited state after the 180° pulse. However, a proportion also ‘swaps’ into the other state, and is not completely refocused (Fig. 2B).

### 2.3 Derivation of the CPMG propagator

Substituting Eq. (21) and its complex conjugate into Eq. (7) allows us to derive an expression for the CPMG propagator *P*:

(32) $$P=\frac{e^{-4\tau_{\mathrm{cp}}R_{2}^{G}}}{{\mathrm{NNN}}^{*}N^{*}}(B_{00}e^{{-\tau }_{\mathrm{cp}}f_{00}}+B_{11}e^{{-\tau }_{\mathrm{cp}}f_{11}})(B_{00}^{*}e^{-\tau_{\mathrm{cp}}f_{00}}+B_{11}^{*}e^{-\tau_{\mathrm{cp}}f_{11}})(B_{00}^{*}e^{-\tau_{\mathrm{cp}}f_{00}}+B_{11}^{*}e^{-\tau_{\mathrm{cp}}f_{11}})(B_{00}e^{{-\tau }_{\mathrm{cp}}f_{00}}+B_{11}e^{{-\tau }_{\mathrm{cp}}f_{11}})$$

This can be simplified by noting that *B*_00_ and *B*_11_ are orthogonal. Secondly, B_xx_^*^B_xx_^*^ = N^*^B_xx_^*^ where *xx* = 00, 11 as the matrices are idempotent. This enables the immediate removal of two of the four terms produced by expanding the central two brackets:

(33) $$P=\frac{e^{-4\tau_{\mathrm{cp}}R_{2}^{G}}}{{\mathrm{NNN}}^{*}}\left(B_{00}e^{-\tau_{\mathrm{cp}}f_{00}}+B_{11}e^{-\tau_{\mathrm{cp}}f_{11}}\right)\left(B_{00}^{*}e^{-2\tau_{\mathrm{cp}}f_{00}^{*}}+B_{11}^{*}e^{-2\tau_{\mathrm{cp}}f_{11}^{*}}\right)\left(B_{00}e^{-\tau_{\mathrm{cp}}f_{00}}+B_{11}{e\text{'}}^{-\tau_{\mathrm{cp}}f_{11}}\right)$$

Physically this corresponds to the fact that there are effectively three free precession periods to consider in the CPMG element of length *τ_cp_*, 2*τ_cp_* and *τ_cp_* respectively in the CPMG element, rather than four, which is implied when two Hahn Echoes are directly concatenated. Expanding Eq. (33) and substituting the triple matrix products of B_xx_B_yy_^*^B_zz_ matrices (*xx*, *yy*, *zz* = 00 or 11) for their complimentary diagonal matrices defined in Eqs. (25) and (29) and frequencies (Eqs. 22):

(34) $$P=\frac{e^{-2\tau_{\mathrm{cp}}(R_{2}^{G}+R_{2}^{E}+k_{\mathrm{ex}})}}{{\mathrm{NNN}}^{*}}\left(\left(\begin{array}{c} C_{\mathrm{st}}^{*}C_{\mathrm{st}}e^{2\tau_{\mathrm{cp}}{∊}_{0}}+\\ -C_{\mathrm{sw}}C_{\mathrm{st}}e^{\tau_{\mathrm{cp}}({∊}_{0}-{∊}_{1})}+\\ C_{\mathrm{st}}C_{\mathrm{sw}}e^{-\tau_{\mathrm{cp}}({∊}_{0}-{∊}_{1})}+\\ -C_{{\mathrm{sw}}^{\prime }}C_{\mathrm{sw}}e^{2\tau_{\mathrm{cp}}{∊}_{1}} \end{array}\right)B_{00}+\left(\begin{array}{c} C_{\mathrm{st}}C_{\mathrm{st}}^{*}e^{-2\tau_{\mathrm{cp}}{∊}_{0}}+\\ C_{\mathrm{st}}^{*}C_{{\mathrm{sw}}^{\prime }}e^{\tau_{\mathrm{cp}}({∊}_{0}-{∊}_{1})}+\\ -C_{{\mathrm{sw}}^{\prime }}C_{\mathrm{st}}^{*}e^{-\tau_{\mathrm{cp}}({∊}_{0}-{∊}_{1})}+\\ -C_{\mathrm{sw}}C_{{\mathrm{sw}}^{\prime }}e^{-2\tau_{\mathrm{cp}}{∊}_{1}} \end{array}\right)B_{11}\right)$$

The products of the ‘stay/stay’ and ‘swap/swap’ matrices have a very simplifying property, which is the motivation for introducing them:

(35) $$\begin{array}{c} C_{\mathrm{st}}C_{\mathrm{st}}^{*}=\left(\begin{array}{cc} P_{\mathrm{st}} & 0\\ 0 & P_{\mathrm{st}}^{*} \end{array}\right)\left(\begin{array}{cc} P_{\mathrm{st}}^{*} & 0\\ 0 & P_{\mathrm{st}} \end{array}\right)=P_{\mathrm{st}}P_{\mathrm{st}}^{*}\left(\begin{array}{cc} 1 & 0\\ 0 & 1 \end{array}\right)\\ C_{\mathrm{sw}}C_{{\mathrm{sw}}^{\prime }}=\left(\begin{array}{cc} P_{\mathrm{sw}} & 0\\ 0 & P_{{\mathrm{sw}}^{\prime }} \end{array}\right)\left(\begin{array}{cc} P_{{\mathrm{sw}}^{\prime }} & 0\\ 0 & P_{\mathrm{sw}} \end{array}\right)=P_{\mathrm{sw}}P_{{\mathrm{sw}}^{\prime }}\left(\begin{array}{cc} 1 & 0\\ 0 & 1 \end{array}\right) \end{array}$$

The products of these matrices amount to multiplication by a constant. Defining:

$$F_{0}=P_{\mathrm{st}}P_{\mathrm{st}}^{*}/{\mathrm{NN}}^{*}=(\Delta \omega^{2}+h_{3}^{2})/{\mathrm{NN}}^{*}$$

(36) $$F_{2}=P_{\mathrm{sw}}P_{{\mathrm{sw}}^{\prime }}/{\mathrm{NN}}^{*}=(\Delta \omega^{2}-h_{4}^{2})/{\mathrm{NN}}^{*}$$

where *F*_0_ *−* *F*_2_ *=* 1, and the normalisation factor ${\mathrm{NN}}^{*}=h_{3}^{2}+h_{4}^{2}$. The propagator then becomes:

(37) $$P=\frac{e^{-2\tau_{\mathrm{cp}}(R_{2}^{G}+R_{2}^{E}+k_{\mathrm{ex}})}}{N}\left((F_{0}e^{2\tau_{\mathrm{cp}}{∊}_{0}}-F_{2}e^{2\tau_{\mathrm{cp}}{∊}_{1}})B_{00}+(F_{0}e^{-2\tau_{\mathrm{cp}}{∊}_{0}}-F_{2}e^{-2\tau_{\mathrm{cp}}{∊}_{1}})B_{11}+(e^{-\tau_{\mathrm{cp}}({∊}_{0}-{∊}_{1})}-e^{\tau_{\mathrm{cp}}({∊}_{0}-{∊}_{1})})(C_{\mathrm{st}}C_{\mathrm{sw}}B_{00}-C_{\mathrm{st}}^{*}C_{{\mathrm{sw}}^{\prime }}B_{11})/{\mathrm{NN}}^{*}\right)$$

The product of the stay/swap matrices do not simplify quite as neatly. Defining:

$$C_{\mathrm{st}}C_{\mathrm{sw}}=\left(\begin{array}{cc} F_{1}^{a} & 0\\ 0 & F_{1}^{b} \end{array}\right)\;\text{and}\;C_{\mathrm{st}}^{*}C_{{\mathrm{sw}}^{\prime }}=\left(\begin{array}{cc} F_{1}^{b} & 0\\ 0 & F_{1}^{a} \end{array}\right)\text{,}\;\text{where}:$$

(38) $$\begin{array}{cc} & F_{1}^{a}=P_{\mathrm{st}}P_{\mathrm{sw}}/{\mathrm{NN}}^{*}=(h_{4}-\Delta \omega )(-{\mathrm{ih}}_{3}-\Delta \omega )/{\mathrm{NN}}^{*}\\ & F_{1}^{b}=P_{\mathrm{st}}^{*}P_{{\mathrm{sw}}^{'}}/{\mathrm{NN}}^{*}=(h_{4}+\Delta \omega )(-{\mathrm{ih}}_{3}+\Delta \omega )/{\mathrm{NN}}^{*} \end{array}$$

where $F_{1}^{a}+F_{1}^{b}=(2\Delta \omega^{2}-{\mathrm{ih}}_{1})/{\mathrm{NN}}^{*}$. These results lead to the definition:

(39) $$B_{01}=C_{\mathrm{sw}}C_{\mathrm{st}}B_{00}-C_{\mathrm{st}}^{*}C_{{\mathrm{sw}}^{\prime }}B_{11}=\left(\begin{array}{cc} F_{1}^{a}O_{E}-F_{1}^{b}O_{G} & (F_{1}^{b}+F_{1}^{a})k_{\mathrm{EG}}\\ (F_{1}^{b}+F_{1}^{a})k_{\mathrm{GE}} & F_{1}^{b}O_{G}-F_{1}^{a}O_{E} \end{array}\right)$$

Noting that $F_{1}^{b}O_{G}=-F_{1}^{a}O_{E}$, proven from Eq. (28), then:

(40) $$B_{01}=\left(\begin{array}{cc} 2F_{1}^{a}O_{E} & (F_{1}^{a}+F_{1}^{b})k_{\mathrm{EG}}\\ (F_{1}^{a}+F_{1}^{b})k_{\mathrm{GE}} & 2F_{1}^{b}O_{G} \end{array}\right)$$

Noting the following four frequencies from Eq. (22), composite frequencies can be defined:

(41) $$\begin{array}{c} E_{0}=2{∊}_{0}=-2(f_{00}^{R}-f_{11}^{R})=2h_{3}\\ E_{2}=2{∊}_{1}=-2i(f_{00}^{I}-f_{11}^{I})=2{\mathrm{ih}}_{4}\\ E_{1}=(E_{0}-E_{2})/2={∊}_{0}-{∊}_{1}=-(f_{00}^{R}-f_{11}^{R})+i(f_{00}^{I}-f_{11}^{I})=h_{3}-{\mathrm{ih}}_{4} \end{array}$$

which leads to an expression for the final CPMG propagator, a central result of this paper, in terms of the matrices *B*_00_, *B*_11_ and *B*_01_, (Eqs. (18) and (40)) the factors *N*, *F*_0_ and *F*_2_ (Eq. (36)) and the frequencies *E*_0_, *E*_1_ and *E*_2_ (Eq. (41)):

(42) $$P=\frac{e^{-2\tau_{\mathrm{cp}}(R_{2}^{G}+R_{2}^{E}+k_{\mathrm{ex}})}}{N}((F_{0}e^{\tau_{\mathrm{cp}}E_{0}}-F_{2}e^{\tau_{\mathrm{cp}}E_{2}})B_{00}+(F_{0}e^{-\tau_{\mathrm{cp}}E_{0}}-F_{2}e^{-\tau_{\mathrm{cp}}E_{2}})B_{11}+(e^{-\tau_{\mathrm{cp}}E_{1}}-e^{\tau_{\mathrm{cp}}E_{1}})B_{01})$$

**Fig. 3:**
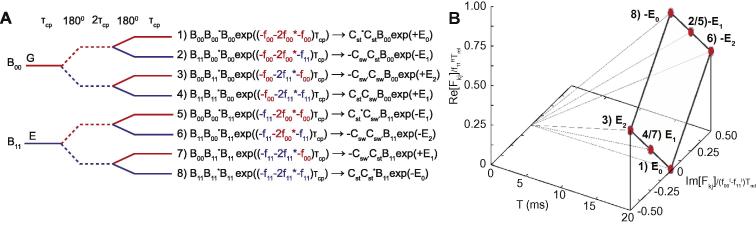
(A) After one Hahn Echo, signal is split into four discrete frequencies (Fig. 2B). When two Hahn Echoes are concatenated to form a single CPMG element, signal is correspondingly split into eight terms (Eq. (42)) with weighting factors that span six unique evolution frequencies (Eq. (41)). Terms that stay in either the ground or excited state ensemble are entirely refocused, and are associated with the purely real evolution frequencies ±*E*_0_. By contrast, the least refocused magnetisation swaps ensemble after both pulses, and can be associated with the imaginary frequencies ±*E*_2_. Between these two cases is the partially refocused magnetisation that evolves at the complex frequencies ±*E*_1_. The terms with their weighting coefficients are illustrated. Note that a factor of *exp*(−2*τ_cp_*(*f*_00_*^R^* + *f_11_^R^*)) has been factorised from the expression, after the arrow. (B) The six frequencies can all be expressed in terms of the difference between either the real part or the imaginary parts of *f*_00_ and *f*_11_, and normalised as explained in the text. The six frequencies give a distinctive geometric pattern when visualised in terms of their real and imaginary components. Here, *T_rel_* = 20 ms. As *f*_11_*^R^* ≫ *f*_00_*^R^*, the term with the lowest relaxation rate is +*E*_0_ that falls at the bottom of the diamond. This term reflects magnetisation that stays in the ground state ensemble for the duration of the element, and dominates the final observed signal.

The coefficients allow physical insight into the types of magnetisation that emerge from a CPMG element (Fig. 3A). Magnetisation takes on one of six discrete evolution frequencies, ±*E*_0_, ±*E*_1_ and ±*E*_2_. Signal that stays with either the ground or excited state ensembles for the duration of the CPMG element is successfully refocused, associated with the factor *F*_0_ and real frequencies ±*E*_0_. By contrast, a portion of the signal effectively swaps from the ground to the excited state twice, once after each 180° pulse. This magnetisation accrues the most net phase, is associated with the factor F_2_, and the imaginary frequencies ±*E*_2_. A further set of signal is associated with swapping at only one of the two 180° pulses, is associated with the matrix *B*_01_ and evolves at the complex frequencies ±*E*_1_. Overall, incoming signal is split into six, each accruing its own phase, ±*E*_0_*τ_cp_*, ±*E*_1_*τ_cp_* or *±E*_2_*τ_cp_*. These frequencies are multiples of each other, and form a distinctive diamond shape when the real and imaginary components are visualised (Fig. 3B).

### 2.4 Derivation of expression for CPMG intensity

To obtain an expression for the CPMG intensity, the CPMG propagator *P* (Eq. (42)) is raised to the power of *N_cyc_*:

(43) $$M=\frac{C}{N}{\left((F_{0}e^{\tau_{\mathrm{cp}}E_{0}}-F_{2}e^{\tau_{\mathrm{cp}}E_{2}})B_{00}+(F_{0}e^{-\tau_{\mathrm{cp}}E_{0}}-F_{2}e^{-\tau_{\mathrm{cp}}E_{2}})B_{11}+(e^{-\tau_{\mathrm{cp}}E_{1}}-e^{\tau_{\mathrm{cp}}E_{1}})B_{01}\right)}^{N_{\mathrm{cyc}}}$$

where *τ_cp_* = *T_rel_*/(4*N_cyc_*) and:

(44) $$C=e^{-T_{\mathrm{rel}}(R_{2}^{G}+R_{2}^{E}+k_{EX})/2}$$

Using the prescription in Eq. (5) and the definitions in Supplementary Section 3, this can be efficiently accomplished by first diagonalising *P*, raising the diagonal elements to the required power of *N_cyc_* and then returning the matrix to the original basis. First the constants required by Eq. (68) are defined, and then placed into Eq. (69). Making use of the trigonometric identities 2 sinh(*x*) = *e^x^* − *e*^−^*^x^* and 2 cosh(*x*) = *e^x^* + *e*^−^*^x^*, and the definitions for *E_x_* (Eq. (41)) and *F_x_* (Eq. (36)):

(45) $$\begin{array}{cc} & v_{1c}=F_{0}\cosh(\tau_{\mathrm{cp}}E_{0})-F_{2}\cosh(\tau_{\mathrm{cp}}E_{2})\\ & v_{1s}=F_{0}\sinh(\tau_{\mathrm{cp}}E_{0})-F_{2}\sinh(\tau_{\mathrm{cp}}E_{2})\\ & v_{2}N=v_{1s}(O_{E}-O_{G})+4O_{E}F_{1}^{a}\sinh(\tau_{\mathrm{cp}}E_{1})\\ & p_{D}N=v_{1s}+(F_{1}^{a}+F_{1}^{b})\sinh(\tau_{\mathrm{cp}}E_{1})\\ & v_{3}={\left(v_{2}^{2}+4k_{\mathrm{EG}}k_{\mathrm{GE}}p_{D}^{2}\right)}^{1/2}\\ & y={\left(\frac{v_{1c}-v_{3}}{v_{1c}+v_{3}}\right)}^{N_{\mathrm{cyc}}} \end{array}$$

Noting that as *E*_2_ is imaginary, cosh(*τ_cp_E*_2_) = cos(*τ_cp_|E*_2_*|*) and sinh(*τ_cp_E*_2_) = *i*sin(*τ_cp_|E*_2_*|*) where the |*x*| denotes complex modulus. The concatenated CPMG elements have the evolution matrix:

(46) $$M=C{\left(v_{1c}+v_{3}\right)}^{N_{\mathrm{cyc}}}\left(\begin{array}{cc} \frac{1}{2}(1+y+\frac{v_{2}}{v_{3}}(1-y)) & \frac{k_{\mathrm{EG}}p_{D}}{v_{3}}(1-y)\\ \frac{k_{\mathrm{GE}}p_{D}}{v_{3}}(1-y) & \frac{1}{2}(1+y-\frac{v_{2}}{v_{3}}(1-y)) \end{array}\right)$$

From Eq. (46) the effective relaxation rate, *R*_2,_*_eff_*, for the ground state magnetisation can be calculated using Eqs. (1), (8), and (46), neglecting the effects of chemical exchange during signal detection (see Supplementary Section 7 for removing this assumption). As *I_G_*(0) = *P_G_*, the central result of the paper is derived, an exact expression for *R*_2,_*_eff_*:

(47) $$R_{2\text{,}\mathrm{eff}}=\frac{R_{2}^{G}+R_{2}^{E}+k_{\mathrm{EX}}}{2}-\frac{N_{\mathrm{cyc}}}{T_{\mathrm{rel}}}\ln(v_{1c}+v_{3})-\frac{1}{T_{\mathrm{rel}}}\ln\left(\frac{1+y}{2}+\frac{1-y}{2v_{3}}(v_{2}+2k_{\mathrm{GE}}p_{D})\right)$$

Finally, as proven in Supplementary Section 4, $v_{3}=\sqrt{v_{1c}-1}$, enabling us to use the identity $\cosh^{-1}(v_{1c})=\text{In}(v_{1c}+\sqrt{v_{1c}^{2}-1})$ and express the result in a simplified form, summarised in Appendix A:

(48) $$R_{2\text{,}\mathrm{eff}}=\frac{R_{2}^{G}+R_{2}^{E}+k_{\mathrm{EX}}}{2}-\frac{N_{\mathrm{cyc}}}{T_{\mathrm{rel}}}\cosh^{-1}(v_{1c})-\frac{1}{T_{\mathrm{rel}}}\ln\left(\frac{1+y}{2}+\frac{1-y}{2\sqrt{v_{1c}^{2}-1}}(v_{2}+2k_{\mathrm{GE}}p_{D})\right)$$

## 3 Comparison to Carver Richards equation

It is interesting to compare this result (Eq. (48)) to the original Carver Richards equation [6]. The explicit relations between our parameters and those in the original work are presented formally in Supplementary Section 4. In terms of present definitions, the Carver Richards equation is:

(49) $$R_{2\text{,}\mathrm{eff}}^{\mathrm{CR}}=\frac{R_{2}^{G}+R_{2}^{E}+k_{\mathrm{EX}}}{2}-\frac{N_{\mathrm{cyc}}}{T_{\mathrm{rel}}}\cosh^{-1}(v_{1c})$$

where the following identity is used to simplify the trigonometric terms [2,42,43]:

$$\cosh^{-1}(F_{0}\cosh(E_{0})-F_{2}\cos(|E_{2}|))=\log({\left(F_{0}\cosh^{2}(E_{0})-F_{2}\cos^{2}(|E_{2}|)\right)}^{1/2}+{\left(F_{0}\sinh^{2}(E_{0})-F_{2}\sin^{2}(|E_{2}|)\right)}^{1/2})$$

The only difference between the precise form described in reference [6] and Eq. (49) is that their free precession delay *τ_cp_* is effectively four times longer. Nevertheless, there are clear similarities between Eqs. (48) and (49), and so the new expression can be expressed as a linear correction to the Carver Richards result, requiring the definitions in Eq. (45):

(50) $$R_{2\text{,}\mathrm{eff}}=R_{2\text{,}\mathrm{eff}}^{\mathrm{CR}}-\frac{1}{T_{\mathrm{rel}}}\ln\left(\frac{1+y}{2}+\frac{1-y}{2\sqrt{v_{1c}^{2}-1}}(v_{2}+2p_{D}k_{\mathrm{GE}})\right)$$

The correction factor is exactly equal to the deviations between the numerical result and the Carver Richards equation described in Fig. 1, to double floating point precision. It is interesting to consider the region of validity of the Carver Richards result. The two results are equal when the correction is zero, which is true when:

(51) $$\sqrt{v_{1c}^{2}-1}\approx v_{2}+2p_{D}k_{\mathrm{GE}}$$

This occurs when *k_GE_p_D_* tends to zero, and so *v*_2_ = *v*_3_. The term *p_D_* is based on the product of the off diagonal elements in the CPMG propagator (Supplementary Section 3). Setting *K_GE_P_D_* to zero amounts to neglecting magnetisation that starts on the ground state ensemble and end on the excited state ensemble and vice versa. This will be a good approximation when *P_G_* ≫ *P_E_*. In practice, significant deviations from the Carver Richards equation can be incurred if *P_E_* > 1% (Fig. 1). Incorporation of the correction term into Eq. (50), summarised in Appendix A, results in an improved description of the CPMG experiment over the Carver Richards equation.

## 4 Determination of $R_{2}^{\infty }$

It is interesting to calculate the effective relaxation rate at high pulsing frequencies. As proven in Supplementary Section 6, in this limit:

(52) $$R_{2\text{,}\mathrm{eff}}^{\infty }=\frac{R_{2}^{G}+R_{2}^{E}+k_{\mathrm{EX}}(1-T)}{2}-\frac{1}{T_{\mathrm{rel}}}\ln\left(\frac{1}{2T}(1+e^{-T_{\mathrm{rel}}k_{\mathrm{EX}}T})\left(T+\tanh\left(\frac{T_{\mathrm{rel}}k_{\mathrm{EX}}T}{2}\right)\left(1+\frac{\Delta R_{2}}{k_{\mathrm{EX}}}\right)\right)\right)$$

where

(53) $$T=\sqrt{2(P_{G}-P_{E})\Delta R/k_{\mathrm{EX}}+{\left(\Delta R/k_{\mathrm{EX}}\right)}^{2}+1}$$

The logarithmic term in Eq. (52) accounts for the duration of the CPMG element. Intuitively, if the duration is less than the timescale of exchange, then additional contributions to the effective relaxation rate will necessarily appear, accounted for by this term. Correspondingly, in the limit *T_rel_k_EX_T* ≫ 1 the logarithmic term is negligible. Going further, in the limit $1\gg 4P_{E}\Delta R_{2}k_{\mathrm{EX}}{\left(k_{\mathrm{EX}}+\Delta R_{2}\right)}^{-2}$ (see Supplementary Section 6), true if *P_E_* is small, or if either *k_EX_* ≫ Δ*R*_2_ or Δ*R*_2_ ≫ *k_EX_*, Eq. (53) can be further simplified, leading to a modified version of Eq. (52):

(54) $$R_{2}^{\infty }=R_{2}^{G}+\frac{P_{E}\Delta R_{2}}{1+\Delta R_{2}/k_{\mathrm{EX}}}$$

Which is identical to the relaxation rate expected for the *R*_1_*_ρ_* experiment in the strong field limit (Ref. [44], *ω*_1_ ≫ *δ_G_*, *δ_E_*, *k_EX_*, Δ*R*_2_, Eqs. (5)–(8)). Thus the fast pulsing limit of the CPMG experiment, and the strong field limit of the *R*_1_*_ρ_* experiment lead to identical relaxation rates, as would be expected. Eq. (54) is similar, but not identical to similarly reported results [2,6]. Going further, when *k_EX_* ≫ Δ*R*_2_ > 0, both the CPMG and *R*_1_*_ρ_* (in the strong field limit) experiments converge on the intuitive population averaged relaxation rate [42]:

(55) limPE→0kex>ΔR2R2∞=PGR2G+PER2E

Finally, in the limit Δ*R*_2_ = 0, the CPMG propagator (Eq. (46)) in the limit of fast pulsing (Eq. (80) using the results in Supplementary Section 1) becomes:

(56) $$M_{\Delta R_{2}=0}^{\infty }=e^{-T_{\mathrm{rel}}R_{2}^{G}}\left(\begin{array}{cc} P_{G} & P_{G}\\ P_{E} & P_{E} \end{array}\right)$$

Which is identical to the evolution matrix for free precession in the limit of fast exchange (Eq. (17) and using the results in Supplementary Section 1). High pulse frequency CPMG experiments only act to make the system appear to be formally in fast exchange limit when Δ*R*_2_ = 0.

## 5 The CPMG experiment as a series expansion

Physical insight into the CPMG experiment is obtained by considering the overall propagator for the CPMG experiment (Eq. (42)), raised to the power *N_cyc_*.

(57) $$M=e^{-2\tau_{\mathrm{cp}}N_{\mathrm{cyc}}(2R_{2}^{G}+f_{00}^{R}+f_{11}^{R})}{\left((F_{0}e^{\tau_{\mathrm{cp}}E_{0}}-F_{2}e^{\tau_{\mathrm{cp}}E_{2}})\frac{B_{00}}{N}+(F_{0}e^{-\tau_{\mathrm{cp}}E_{0}}-F_{2}e^{-\tau_{\mathrm{cp}}E_{2}})\frac{B_{11}}{N}+(e^{-\tau_{\mathrm{cp}}E_{1}}-e^{\tau_{\mathrm{cp}}E_{1}})\frac{B_{01}}{N}\right)}^{N_{\mathrm{cyc}}}$$

The CPMG experiment can be considered in terms of a series expansion. The propagator initially contains six unequally weighted evolution frequencies, ±*E*_0_, ±*E*_1_ and ±*E*_2_, where the cofactors are the product of an *F_x_* (*x* = 0, 2) constant, (Eq. (36)), and a *B_xx_* (*xx* = 00, 11, 01) matrix (Eqs. (18) and (40)). Raising these terms to the power *N_cyc_* will result in new terms that can be represented in terms of sums and differences of the six frequencies, and weighting coefficients. Temporarily ignoring the coefficients, the frequencies that can be involved in the expansion can be revealed using Eq. (41), noting that *ε*_0_ is real and *ε*_1_ is imaginary:

(58) $${\left(e^{t_{\mathrm{cp}}2{∊}_{0}}+e^{t_{\mathrm{cp}}2{∊}_{1}}+e^{-t_{\mathrm{cp}}2{∊}_{0}}+e^{-t_{\mathrm{cp}}2{∊}_{1}}+e^{-t_{\mathrm{cp}}({∊}_{0}+{∊}_{1})}+e^{t_{\mathrm{cp}}({∊}_{0}+{∊}_{1})}\right)}^{N_{\mathrm{cyc}}}={\left(e^{t_{\mathrm{cp}}({∊}_{0}+{∊}_{1})}+e^{-t_{\mathrm{cp}}({∊}_{0}+{∊}_{1})}\right)}^{N_{\mathrm{cyc}}}{\left(e^{t_{\mathrm{cp}}({∊}_{0}-{∊}_{1})}+1+e^{-t_{\mathrm{cp}}({∊}_{0}-{∊}_{1})}\right)}^{N_{\mathrm{cyc}}}$$

**Fig. 4:**
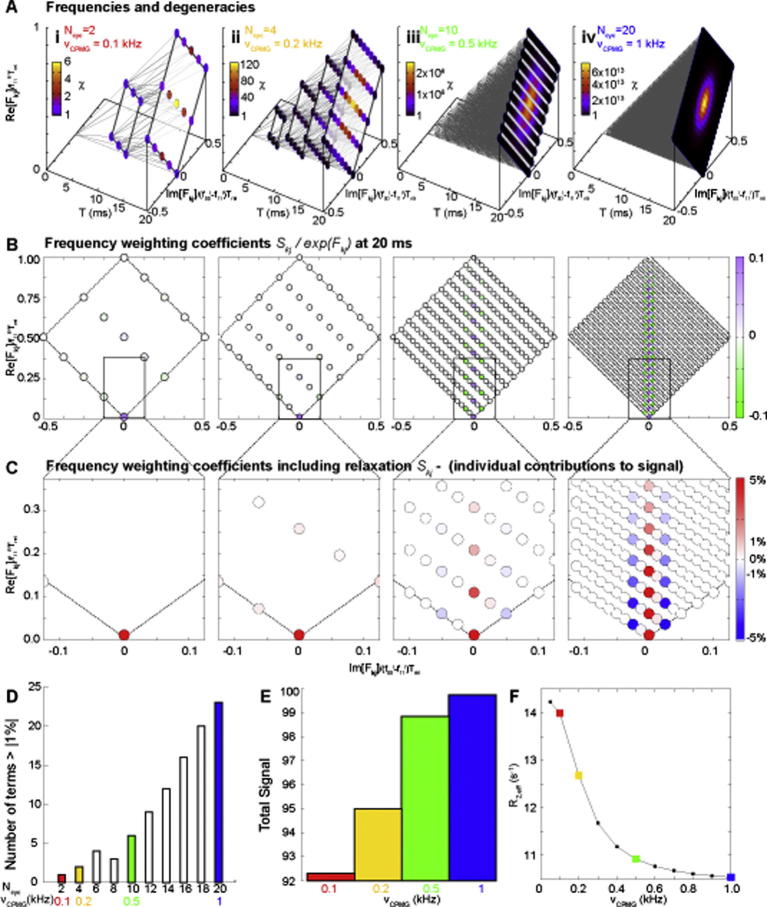
The CPMG experiment in terms of the evolution of discrete frequencies. The following are simulated with the following exchange parameters: Δ*ω* = 1 ppm, Lamor frequency 200 MHz, *k_EG_* = 400 s^−1^, *k_GE_* = 5 s^−1^, *P_E_* = 1.2%, *R*_2_*^G^* = 10 s^−1^, *R*_2_*^E^* = 50 s^−1^*T_rel_* = 20 ms, *N_cyc_* = 1, 2, 4, 6, 8, 10, 12, 14, 16, 18, 20, corresponding to *v_CPMG_* frequencies in the range 50 Hz to 1 kHz. (A) Continued from Fig. 3B, as the number of CPMG elements increases, so too does the number of discrete frequencies. In a constant time CPMG experiment, the range the frequencies at the end of the experiment is the same for all values of *N_cyc_*. The spectral resolution, the density of individual observed frequencies, therefore, increases with increasing *N_cyc_*. Due to the combinatorial nature of the way the individual frequencies emerge, the number of pathways that lead to a given frequency, *χ*, increases massively with increasing *N_cyc_*, leading to a large combinatorial weighting favouring central terms. (B) Each frequency has a unique weighting. The effective weighting constant, neglecting relaxation (Eq. (63)/*exp*(*F*(*k*, *j*)), is shown for each frequency for the given exchange parameters. The combinatorial factor *χ* acts to overcome initially low weighting coefficients, increasing the probability that the central terms will contribute to signal intensity at high values of *N_cyc_*. (C) In order to calculate the individual contribution of each frequency to the final signal (Eq. (63)), the weighting coefficient in B must be multiplied by the appropriate relaxation rate (*exp*(position-on-the-*y*-axis)). As higher values of *y* have significantly higher relaxation rates, terms with large weighting coefficients in B end up presenting only a small contribution to the overall signal intensity. (D) Taken together, the number of terms whose magnitude is greater than 1% (Eq. (63)) increases with *N_cyc_*. (E) The total intensity of the observed resonance, as obtained by summing over all terms in C. (F) The corresponding relaxation dispersion curve, as typically measured in a CPMG experiment, where intensities are converted to a relaxation rate using Eq. (1).

The expansion results therefore in the product of a binomial expansion over *τ_cp_*(*ε*_0_ + *ε*_1_), and a trinomial expansion over *τ_cp_*(*ε*_0_ − *ε*_1_). The expansion in Eq. (57) will therefore result in 3*^Ncyc^*2*^Ncyc^* individual terms, arranged over (1 + *N_cyc_*)(1 + 2*N_cyc_*) possible frequencies (Fig. 4A). Including the average relaxation rate factor at the front of Eq. (57), 2*τ_cp_N_cyc_*(*f*_00_*^R^* + *f*_11_*^R^*), the real part of the frequencies will fall between 4*N_cyc_τ_cp_f*_00_*^R^* and 4*N_cyc_τ_cp_f*_11_*^R^*, or *T_rel_f*_00_*^R^* to *T_rel_f*_11_*^R^*. These two real limiting frequency values correspond to magnetisation that stays in either the ground state, or excited state for the duration of the CPMG experiment. Similarly, the imaginary component varies from −2*τ_cp_N_cyc_ε*_1_ to 2*τ_cp_N_cyc_ε*_1_, which can be expressed as ±*T_rel_*(*f*_00_*^I^* *−* *f*_11_*^I^*)/2. The two imaginary limiting values correspond to magnetisation that ‘swaps’ ensembles after each 180° pulse, spending equal time in the ground and excited state ensembles. The imaginary limiting values correspond to the least refocused magnetisation. All four frequency limits are proportional to *T_rel_*. This provides a strong justification for performing constant time CPMG experiments, as this means that the relaxation for each term, and the maximum phase that any one term can accrue will be constant for all values of *N_cyc_*. The complete set of discrete frequencies that can potentially contribute to the signal intensity, parameterised in terms of the indices *j* and *k*:

(59) $$F_{k\text{,}j}=\left(\left(\frac{k-2j+1}{N_{\mathrm{cyc}}}\right)\left(f_{11}^{R}-f_{00}^{R}\right)+2\left(f_{00}^{R}+f_{11}^{R}\right)+i\left(\frac{-k-2j+3}{N_{\mathrm{cyc}}}+2\right)\left(f_{00}^{I}-f_{11}^{I}\right)\right)\frac{T_{\mathrm{rel}}}{4}$$

with the index *k* running from 1 to 1 + 2*N_cyc_* describing the trinomial expansion in *ε*_0_ − *ε*_1_, and *j* running from 1 to 1 + *N_cyc_* describing the binomial expansion in *ε*_0_ + *ε*_1_. The geometric distribution of these the real and imaginary components of these frequencies is illustrated in Figs. 3B and 4A, where the real component has been normalised by a factor of *f*_11_*^R^T_rel_*, and the imaginary terms by (*f*_00_*^I^* *−* *f*_11_*^I^*)*T_rel_*. Using these normalisations, the range of frequencies are independent on *N_cyc_* and take the form of a diamond with limits in the imaginary dimension of (−0.5, 0.5) and in the real dimension of (*f*_00_*^R^*/*f*_11_*^R^*) to 1. As *f*_00_*^R^* ≪ *f*_11_*^R^*, on this scale the first term appears to be very close to zero, and the terms ‘higher’ up the diamond on the real axis have significantly larger relaxation rates. In the constant time CPMG experiment, the range of the resolvable frequencies is identical. The spectral resolution is limited by the density of frequencies which increases substantially with increasing *N_cyc_* (Fig. 4A).

The simultaneous binomial and trinomial expansions result in there being many different pathways that can lead to the same final net evolution frequency. The total number of individual pathways that will contribute at each frequency is given by the product of the coefficients of the two series, written here in terms of the Gamma function, a generalisation of the factorial, Γ(x+1)=x!:

(60) $$\chi_{k\text{,}j}=\chi_{k\text{,}j}^{\mathrm{bi}}\chi_{k\text{,}j}^{\mathrm{tri}}=\left(\frac{\Gamma (n+1)}{\Gamma (n-k+1)\Gamma (k+1)}\right)\left(\overset{n}{\underset{j=0}{\sum }}\frac{\Gamma (n+1)}{\Gamma (j+k+1)\Gamma (n-2j-k+1)}\right)$$

The degeneracies of each frequency are strongly dependent on *N_cyc_*. Initially, each of the six frequencies has equal degeneracy (*N_cyc_* = 1, Fig. 3B). At successively higher values of *N_cyc_*, there exists a strong combinatorial preference for terms to converge on the central frequency (Fig. 4A). This combinatorial factor effectively describes the additional mixing between ground and excited ensembles that occur at increased *ν_CPMG_*. It is important to note however that the frequencies emerging from the CPMG block are not equally weighted, and using Eq. (59), the CPMG propagator can be expressed as follows:

(61) $$M=e^{-T_{\mathrm{rel}}R_{2}^{G}}\overset{1+2N_{\mathrm{cyc}}}{\underset{k=1}{\sum }}\overset{1+N_{\mathrm{cyc}}}{\underset{j=1}{\sum }}B_{k\text{,}j}\exp(F_{k\text{,}j})$$

where

(62) $$B_{k\text{,}j}=\overset{\chi_{k\text{,}j}}{\underset{a=1}{\sum }}\overset{N_{\mathrm{cyc}}}{\underset{b=1}{\prod }}\frac{F_{x}B_{\mathrm{yy}}}{N}$$

with *x* equal to 0, 1 or 2, and *yy* can equal either 00, 11 or 01 depending on the history of each term. Each term will be a product of *N_cyc_* individual *F_x_B_yy_* factors, and the sum is over all terms with the same frequency, *F_kj_*. As the matrix multiplication depends on the order with which the matrices are multiplied, these factors are evaluated numerically in what follows. Neglecting chemical exchange during signal acquisition (Supplementary Section 7), the overall ground state signal intensity obtained after a CPMG experiment will be given by Eq. (8). Using a combination of Eqs. (8) and (61) the individual contribution of each frequency at a given *k* and *j*, to the overall signal of the observed ground state resonance can be calculated from:

(63) $$S_{\mathrm{kj}}=\frac{I_{\mathrm{kj}}}{I(0)}=\left(B_{\mathrm{kj}}(0\text{,}0)+B_{\mathrm{kj}}(0\text{,}1)\frac{P_{E}}{P_{G}}\right)e^{F_{\mathrm{kj}}}$$

The individual term coefficients are shown in Fig. 4B for the given exchange parameters, temporarily neglecting relaxation effects from the exponential term *exp*(*F_kj_*).

At higher pulsing frequencies therefore, the combinatorial factors inherent to the experiment considerably increase the influence of frequencies that correspond to mixtures of ground and excited state ensembles (Fig. 4A). When the relaxation inherent in the exponential term is included, the contribution from the terms that have spent more time on the excited state is heavily attenuated, as *f*_11_*^R^* ≫ *f*_00_*^R^* (Fig. 4C, terms higher up the *y*-axis). Nevertheless, as more frequency terms contribute to the signal (Fig. 4C and D), and the observed intensity increases (Fig. 4E) leading to the characteristic form of the CPMG curve (Fig. 4F). In summary, the combinatorial factors associated with pathway degeneracy (Fig. 4A) tend to favour these terms as the fast pulsing limit is approached. This leads to magnetisation that would effectively have otherwise have decayed away to nothing in the low pulsing frequency, to instead be converted to observable signal (Fig. 4E and F). As a consequence, faster pulsing leads to greater signal intensity over the same constant time.

It is common to describe the action of the CPMG experiment in terms of its ability to refocus magnetisation. Here it is shown that this is an incomplete physical description. The CPMG experiment does tend to refocus chemical shift as expected, but it is only refocused magnetisation that spends the majority of its time in the ground state mixed ensemble (associated with the frequency *f*_00_) that relaxes sufficiently slowly to contribute significantly to the observed signal. At low pulsing frequencies, only magnetisation that remains with the ground state ensemble contributes significantly to signal intensity. By contrast, at higher pulsing frequencies, the ground and excited mixed-state ensembles are interconverted, enabling new pathways for magnetisation to follow. A number of these pathways are associated with spending only a relatively small amount of time on the excited state. Magnetisation that passes down these pathways is consequently sufficiently long lived that it can contribute to the observed signal, rather than relaxing away to nothing. It is this slowly relaxing magnetisation that can lead to the increase in signal intensity that is characteristic of a CPMG relaxation dispersion experiment. Quantitative analysis of the variance of signal intensity with CPMG pulsing frequency can therefore then yield insights into the chemical process that underlies the exchange in the system under study.

## 6 Conclusion

An exact solution describing how the effective transverse relaxation rate varies as a function of CPMG pulse frequency is presented (Eq. (50), summarised in Appendix A). This expression takes the form of a linear correction to the widely used Carver Richards equation [6]. Expressions are provided that take into account exchange during signal detection (Eqs. (90) and (91)) [41], enabling an improved theoretical description of the CPMG experiment suitable for data analysis. The formula provides a ca. 130× speed up in calculation of CPMG data over numerical approaches, and is both faster and requires a lower level of precision to provide exact results than already existing approaches (Supplementary Section 8). Freely downloadable versions in C and python are available for download as described in Appendix A. As this expression is exactly differentiable it has the potential to greatly speed up fitting to experimental data. It is important to note that effects of off resonance [40] and finite time 180° pulses [39] will lead to deviations from ideality [25,28]. Moreover, additional spin-physics such as scalar coupling and differential relaxation are neglected in this approach. In the case of experiments where in-phase magnetisation is created, heteronuclear decoupling is applied during the CPMG period [25,28], and CPMG pulses are applied on-resonance, the formula will be in closest agreement with experimental data. All of these additional effects are readily incorporated into a numerical approach [32], which will give the most complete description of the experiment. The formula retains value however in offering both the potential to provide fast initial estimates for such algorithms, and in providing insight into the physical principles behind the experiment.

## Figures and Tables

**Table:** 

*(i) Establish the complex free precession Eigenfrequency*	
$\begin{array}{c} h_{1}=2\Delta \omega (\Delta R_{2}+k_{\mathrm{EG}}-k_{\mathrm{GE}})\\ h_{2}={\left(2\Delta R_{2}+k_{\mathrm{EG}}-k_{\mathrm{GE}}\right)}^{2}+4k_{\mathrm{EG}}k_{\mathrm{GE}}-\Delta \omega^{2} \end{array}$	$f_{00}=\frac{1}{2}(\Delta R_{2}+k_{\mathrm{EX}}-h_{3})+\frac{i}{2}(\Delta \omega -h_{4})$
$\begin{array}{c} h_{3}=\frac{1}{\sqrt{2}}\sqrt{h_{2}+\sqrt{h_{1}^{2}+h_{2}^{2}}}\\ h_{4}=\frac{1}{\sqrt{2}}\sqrt{-h_{2}+\sqrt{h_{1}^{2}+h_{2}^{2}}} \end{array}$	The ground state ensemble evolution frequency *f*_00_ expressed in separated real and imaginary components, in terms of definitions h_1__–__4_. (Eqs. (11)–(14)). Δ*R*_2_ = *R*_2_*_E_* *−* *R*_2_*_G_*

*(ii) Define substitutions for ‘stay’ and ‘swap’ factors*	
$\begin{array}{c} N=h_{3}+{\mathrm{ih}}_{4}\\ {\mathrm{NN}}^{*}=h_{3}^{2}+h_{4}^{2} \end{array}$	Weighting factors for frequencies (*E*_0–2_) emerging from a single CPMG block, *F*_0–2_. (Eqs. (36), (38), and (41)).
$\begin{array}{c} F_{0}=(\Delta \omega^{2}+h_{3}^{2})/{\mathrm{NN}}^{*}\\ F_{2}=(\Delta \omega^{2}-h_{4}^{2})/{\mathrm{NN}}^{*}\\ F_{1}^{b}=(h_{4}+\Delta \omega )(\Delta \omega -{\mathrm{ih}}_{3})/{\mathrm{NN}}^{*} \end{array}$	$\begin{array}{c} E_{0}=2h_{3}\\ |E_{2}|=2h_{4}\\ E_{1}=h_{3}-{\mathrm{ih}}_{4} \end{array}$

*(iii) The Carver Richard’s result* (Eq. (49))	
$v_{1c}=F_{0}\cosh(\tau_{\mathrm{cp}}E_{0})-F_{2}\cos(\tau_{\mathrm{cp}}|E_{2}|)$	$R_{2\text{,}\mathrm{eff}}^{\mathrm{CR}}=\frac{R_{2}^{G}+R_{2}^{E}+k_{\mathrm{EX}}}{2}-\frac{N_{\mathrm{cyc}}}{T_{\mathrm{rel}}}\cosh^{-1}(v_{1c})$

*(iv) Final result*	
$\begin{array}{c} v_{1s}=F_{0}\sinh(\tau_{\mathrm{cp}}E_{0})-{\mathrm{iF}}_{2}\sin(\tau_{\mathrm{cp}}|E_{2}|)\\ v_{4}=-2(k_{\mathrm{GE}}-f_{00})F_{1}^{b}+k_{\mathrm{GE}}(2\Delta \omega^{2}-{\mathrm{ih}}_{1})/{\mathrm{NN}}^{*}\\ v_{5}=(\Delta R_{2}+k_{\mathrm{EX}}+i\Delta \omega )v_{1s}-2v_{4}\sinh(\tau_{\mathrm{cp}}E_{1}) \end{array}$	$y={\left(\frac{v_{1c}-{\left(v_{1c}^{2}-1\right)}^{1/2}}{v_{1c}+{\left(v_{1c}^{2}-1\right)}^{1/2}}\right)}^{N_{\mathrm{cyc}}}$
$R_{2\text{,}\mathrm{eff}}=R_{2\text{,}\mathrm{eff}}^{\mathrm{CR}}-\frac{1}{T_{\mathrm{rel}}}\ln\left(\frac{1+y}{2}+\frac{(1-y)v_{5}}{2N\sqrt{v_{1c}^{2}-1}}\right)$	Four identities to assist efficient matrix exponentiation optimised for numerical calculation, and the final result. Modified from Eqs. (45) and (50)

## Appendix A Recipe for exact calculation of *R*_2,_*_eff_*

A complete summary of all steps required to calculate *R*_2,_*_eff_* from *R*_2_*_E_*, *R*_2_*_G_*, Δ*ω*, *k_ex_* and *P_E_*, *N_cyc_* and *T_rel_*. An implementation of the result written in python can be downloaded from http://baldwinlab.chem.ox.ac.uk/resources.

## Appendix B Supplementary material

Supplementary data 1 Supplementary Sections 1–8, containing equations 64–91 are available online. The sections are titled 1 – The evolution frequencies in the fast and slow exchange limits, 2 – Diagonalising an idempotent product of matricies, 3 – Raising a matrix to a power, 4 – Relation to Carver Richards equation, 5 – Derivation of $v_{3}^{2}=v_{1}^{2}-1$, 6 – Determination of $R_{2}^{\infty }$, 7 – Correction for exchange during indirect evolution periods. 8 – Comparison to existing algorithms.
