# Supplementary material for: An exact solution for R2,eff in CPMG experiments in the case of two site chemical exchange
Source: J Magn Reson. 2014 Jul;244(100):114–24. doi: 10.1016/j.jmr.2014.02.023 (PMC4067747; doi:10.1016/j.jmr.2014.02.023)
Supplement: Supplementary data 1 — Supplementary Sections 1–8, containing equations 64–91 are available online. The sections are titled 1 – The evolution frequencies in the fast and slow exchange limits, 2 – Diagonalising an idempotent product of matricies, 3 – Raising a matrix to a power, 4 – Relation to Carver Richards equation, 5 – Derivation of v32=v12-1, 6 – Determination of R2∞, 7 – Correction for exchange during indirect evolution periods. 8 – Comparison to existing algorithms. [file mmc1.pdf]

Supplementary information for:

An exact solution for $R_{2,eff}$ in CPMG experiments in the case of two site chemical exchange

Andrew J. Baldwin[1]

1. Physical and Theoretical Chemistry Laboratory, University of Oxford, Oxford, UK, OX1 3QZ

Correspondence to be addressed to andrew.baldwin@chem.ox.ac.uk

Supplementary Section 1 – The evolution frequencies in the fast and slow exchange limits

In both the fast and slow exchange limits, h_2>>h_1. This enables us to simplify h_3 and h_4 and obtain expressions for the real and imaginary parts of the free precession frequencies. When using the series expansion $(1+x)^n=1+nx$, care must be taken to note the magnitude of the larger component, h_2. Limiting expressions for h_3 and h_4 can be obtained from:

$$h_{+/-}=\frac{1}{\sqrt{2}}\sqrt{\pm h_2+|h_2|+\frac{h_1^2}{2|h_2|}}$$

Where + corresponds to h_3 and – corresponds to h_4. Defining:

$$z_1=\left(\Delta R_2^2+2\Delta R_2(P_G-P_E)k_{EX}+k_{EX}^2\right)^{1/2}$$

Enables us to recast h_1 and h_2:

$$h_1=2\Delta\omega\left(z_1^2-4P_GP_Ek_{EX}^2\right)^{1/2}$$
$$h_2=z_1^2-\Delta\omega^2$$

And so $h_2^{fast}=z_1^2$ and $h_2^{slow}=-\Delta\omega^2$. In the limit $\Delta R_2=0$, $h_2^{fast}=z_1^2=k_{EX}^2$ and $h_1=2\Delta\omega k_{EX}^2(P_G-P_E)$. Taking care with the sign of h_2, which changes between the fast and slow limits:

$$h_3^{fast}=h_4^{slow}=|h_2|^{1/2}+\frac{h_1^2}{8|h_2|^{3/2}}=|z_1^2-\Delta\omega^2|^{1/2}+\frac{\Delta\omega^2\left(z_1^2-4P_GP_Ek_{EX}^2\right)}{2|z_1^2-\Delta\omega^2|^{3/2}}$$
$$h_3^{slow}=h_4^{fast}=\frac{h_1}{2|h_2|^{1/2}}=\frac{\Delta\omega\left(z_1^2-4P_GP_Ek_{EX}^2\right)^{1/2}}{|z_1^2-\Delta\omega^2|^{1/2}}$$

A general definition for fast and slow exchange when $\Delta R!=0$ is z_1>>$\Delta\omega$, and $\Delta\omega$>>z_1 respectively. Applying this definition leads to the following limits, with $\Delta R_2!=0$ (left) and $\Delta R_2=0$ (right):

$$\begin{aligned}h_3^{fast}&=|z_1|-\frac{2\Delta\omega^2P_GP_Ek_{EX}^2}{|z_1|^3}\\ h_4^{slow}&=|\Delta\omega|-\frac{2P_GP_Ek_{EX}^2}{|\Delta\omega|}\\ h_3^{slow}&=\left(z_1^2-4P_GP_Ek_{EX}^2\right)^{1/2}\\ h_4^{fast}&=\frac{\Delta\omega\left(z_1^2-4P_GP_Ek_{EX}^2\right)^{1/2}}{|z_1|}\end{aligned}\qquad(64)$$

$$\begin{aligned}h_3^{fast}(\Delta R_2=0)&=k_{EX}-\frac{2P_GP_E\Delta\omega^2}{k_{EX}}\\ h_4^{slow}(\Delta R_2=0)&=|\Delta\omega|-\frac{2P_GP_Ek_{EX}^2}{|\Delta\omega|}\\ h_3^{slow}(\Delta R_2=0)&=k_{EX}\left(P_G-P_E\right)\\ h_4^{fast}(\Delta R_2=0)&=\Delta\omega\left(P_G-P_E\right)\end{aligned}\qquad(65)$$

These expressions can be used to obtain limiting values for the free precession frequencies when used in conjunction with equation (14). The exchange-induced shift of the ground state due to chemical exchange for example is given by $\Delta\omega - h_4$. In the fast exchange limit when $k_{EX} >> \Delta R$, this is equal to $\Delta\omega P_E$.

Supplementary Section 2. Diagonalising an idempotent product of matricies

A diagonal matrix C is sought such that

$$A \cdot B = C \cdot B$$

If the matrices are expanded in terms of their coefficients:

$$A = \begin{pmatrix} a_{00} & a_{01} \\ a_{10} & a_{11} \end{pmatrix}, B = \begin{pmatrix} b_{00} & b_{01} \\ b_{10} & b_{11} \end{pmatrix}, C = \begin{pmatrix} c_{00} & 0 \\ 0 & c_{11} \end{pmatrix}$$

Then the original equality can be expressed:

$$\begin{pmatrix} a_{00}b_{00} + a_{01}b_{10} & a_{00}b_{01} + a_{01}b_{11} \\ a_{10}b_{00} + a_{11}b_{10} & a_{10}b_{01} + a_{11}b_{11} \end{pmatrix} = \begin{pmatrix} c_{00}b_{00} & c_{00}b_{01} \\ c_{11}b_{10} & c_{11}b_{11} \end{pmatrix}$$

Leading to definitions of the two diagonal coefficients for C:

$$\begin{aligned} c_{00} &= a_{00} + a_{01}\frac{b_{10}}{b_{00}} = a_{00} + a_{01}\frac{b_{11}}{b_{01}} \\ c_{11} &= a_{11} + a_{10}\frac{b_{00}}{b_{10}} = a_{11} + a_{10}\frac{b_{01}}{b_{11}} \end{aligned} \qquad (66)$$

It is possible to perform this operation when $b_{00}b_{11} = b_{01}b_{10}$, as is the case for matrices B_{00} and B_{11}.

Supplementary Section 3. Raising a matrix to a power

A matrix P can be taken to an arbitrary power if it is first diagonalised it and then:

$$M = P^N = JP_D^N J^{-1} \qquad (67)$$

Where P_D is the diagonal matrix of Eigenvalues and J is the matrix of eigenvectors. Using the following identities, noting that p(i,j) gives the ith row and the jth column of P:

$$
\begin{aligned}
v_1 &= P(0,0) + P(1,1) \\
v_2 &= P(0,0) - P(1,1) \\
v_3 &= \left(v_2^2 + 4P(0,1)P(1,0)\right)^{1/2} \\
y &= \left(\frac{v_1 - v_3}{v_1 + v_3}\right)^{N_{cyc}}
\end{aligned} \tag{68}
$$

Yielding the following:

$$
M = \left(\frac{v_1}{2} + \frac{v_3}{2}\right)^{N_{cyc}} \begin{pmatrix} \frac{1}{2}\left(1 + y + \frac{v_2}{v_3}(1-y)\right) & \frac{(1-y)P(0,1)}{v_3} \\ \frac{(1-y)P(1,0)}{v_3} & \frac{1}{2}\left(1 + y - \frac{v_2}{v_3}(1-y)\right) \end{pmatrix} \tag{69}
$$

Supplementary Section 4. Relation to Carver Richards equation

The Carver Richards equation uses several identities (the 'A' refers to references in the original work). These are directly related to identities used in this work, which are explicitly re-defined here. The Carver Richards equation is precisely correct if their definition of τ_{cp} is four times the value that used in this paper [6]:

$$\tau_{CP} = \frac{1}{4\nu_{CPMG}} \qquad \text{Eq A8}$$

$$\alpha_- = -(\Delta R_2 - k_{GE} + k_{EG})$$

$$\zeta = 2\Delta\omega\alpha_- = -h_1 \qquad \text{Eq A7}$$

$$\Psi = \alpha_-^2 + 4k_{EG}k_{GE} - \Delta\omega^2 = h_2 \qquad \text{Eq A6}$$

$$\xi = \frac{2\tau_{CP}}{\sqrt{2}}\sqrt{\Psi + \sqrt{\Psi^2 + \zeta^2}} = 2h_3\tau_{CP} = \tau_{cp}E_0 \qquad \text{Eq A4}$$

$$\eta = \frac{2\tau_{CP}}{\sqrt{2}}\sqrt{-\Psi + \sqrt{\Psi^2 + \zeta^2}} = 2h_4\tau_{CP} = \tau_{cp}E_2 \qquad \text{Eq A5}$$

$$D_+ = \frac{1}{2}\left(1 + \frac{\Psi + 2\Delta\omega^2}{\sqrt{\Psi^2 + \zeta^2}}\right) = F_0 \qquad \text{Eq A3}$$

$$D_- = \frac{1}{2}\left(-1 + \frac{\Psi + 2\Delta\omega^2}{\sqrt{\Psi^2 + \zeta^2}}\right) = F_2$$

(70)

These gives the following expression for $R_{2,eff}$:

$$R_{2,eff}^{CR} = \frac{R_2^G + R_2^E + k_{EX}}{2} - \frac{1}{4\tau_{cp}} \cosh^{-1}\left(D_+ \cosh\xi - D_- \cos\eta\right) \tag{71}$$

Our derivation enables physical meaning to be assigned to these constants. ξ and η are differences of the real and imaginary components of the free precession frequencies f_{00} and f_{11} (equation (41)), D_+ and D_- are the stay/stay (F_0), and swap/swap (F_2) coefficients (equation (36)) and ζ and ψ are parameters that enable the free precession frequencies to be written in a more concise form (equation (12)). Note that in reference [2], in equation 25, the definition used for τ_{cp} is twice that used in this work but is otherwise identical.

Supplementary Section 5. Derivation of $v_3^2 = v_1^2 - 1$

Noting the definitions in equation (45), as a starting point, v_3 can be expanded:

$$v_3^2 = v_2^2 + 4k_{EG}k_{GE}p_D^2 \tag{72}$$

Which can be expanded to reveal:

$$v_3^2 = A/N^2\left(F_0\sinh(\tau_{cp}E_0) - F_2\sinh(\tau_{cp}E_2)\right)^2 + B/N^2\sinh^2(\tau_{cp}E_1) \tag{73}$$

Where:

$$\begin{aligned} A &= \left(O_E - O_G\right)^2 + 4k_{GE}k_{EG} = N^2 \\ B &= 4\left(\left(F_1^a + F_1^b\right)k_{EG}k_{GE} + 4\left(O_E F_1^a\right)^2\right) = -4F_0F_2N^2 \end{aligned} \tag{74}$$

A term of the form $C\sinh(\tau_{cp}E_1)\left(F_0\sinh(\tau_{cp}E_0) - F_2\sinh(\tau_{cp}E_2)\right)$ also appears, although:

$$C = 8(O_E - O_G)O_G F_1^b - 8k_{EG}k_{GE}\left(F_1^a + F_1^b\right) = 0 \tag{75}$$

Combining equations (73) and (74):

$$v_3^2 = \left(F_0\sin(\tau_{cp}E_0) - F_2\sinh(\tau_{cp}E_2)\right)^2 - 4F_0F_2\sinh(\tau_{cp}E_1)^2 \tag{76}$$

By noting that $\sinh(\tau_{cp}E_1)^2 = (\cosh(\tau_{cp}(E_0 - E_2)) - 1)/2$,

$\cosh x \cosh y + \sinh x \sinh y = \cosh(x+y)$, $\sin^2 x + \cos^2 x = 1$, $\cosh^2 x - \sinh^2 x = 1$,

$\sinh(ix) = i\sin(x)$, $\cosh(ix) = \cos(x)$ and $F_0 - F_2 = 1$, the following identity can be proven:

$$\left(F_0 \sin(\tau_{cp}E_0) - F_2 \sinh(\tau_{cp}E_2)\right)^2 - 4F_0F_2 \sinh(\tau_{cp}E_1)^2 = \left(F_0 \cosh(\tau_{cp}E_0) - F_2 \cosh(\tau_{cp}E_2)\right)^2 - 1$$

From which it follows that:

$$v_3^2 = v_{1c}^2 - 1 \tag{77}$$

Equation (50) is therefore is exact, and is accurate as the assumptions that lead to its derivation.

Note that this derivation is equivalent to proving 4*P(0,0)P(1,1)-1=P(0,1)P(1,0)* from equation (42).

Supplementary Section 6. Determination of R_2^{∞}

When N_{cyc} becomes large, the limit of small τ_{cp}, both cosh$(\tau_{cp}x)$ and cos$(\tau_{cp}x)$ tend to $1+(\tau_{cp}x)^2/2$, and the terms in sinh$(\tau_{cp}x)$ and sin$(\tau_{cp}x)$ tend to $\tau_{cp}x$, and $p_D=2\tau_{cp}$. Defining:

$$v_{\pm} = \left(v_1 + v_3\right)^{N_{cyc}} \pm \left(v_1 - v_3\right)^{N_{cyc}} \tag{78}$$

And noting that at high N_{cyc}, $1 >> v_3 >> v_1$ and noting that $(1+a/x)^x$ tends to exp(a) as x tends to infinity:

$$v_{\pm} \approx \exp\left(N_{cyc}\left(v_1 - 1\right)\right)\left(\exp\left(N_{cyc}v_3\right) \pm \exp\left(-N_{cyc}v_3\right)\right) \tag{79}$$

Consequently, the propagator for the CPMG element from equation (46) can be written:

$$M^{\infty} = C\begin{pmatrix} \frac{1}{2}\left(v_+ + \frac{v_2v_-}{v_3}\right) & \frac{2\tau_{cp}k_{EG}v_-}{v_3} \\ \frac{2\tau_{cp}k_{GE}v_-}{v_3} & \frac{1}{2}\left(v_+ - \frac{v_2v_-}{v_3}\right) \end{pmatrix} \tag{80}$$

Noting that $v_{\pm}$ can be expressed in terms of hyperbolic trigonometric functions:

$$M^{\infty} = Ce^{N_{cyc}(v_1-1)}e^{N_{cyc}v_3}\left(1+e^{-2N_{cyc}v_3}\right)\begin{pmatrix} \frac{1}{2}\left(1+\tanh\left(N_{cyc}v_3\right)\frac{v_2}{v_3}\right) & \tanh\left(N_{cyc}v_3\right)\frac{2\tau_{cp}k_{EG}}{v_3} \\ \tanh\left(N_{cyc}v_3\right)\frac{2\tau_{cp}k_{GE}}{v_3} & \frac{1}{2}\left(1-\tanh\left(N_{cyc}v_3\right)\frac{v_2}{v_3}\right) \end{pmatrix} \tag{81}$$

Leading to a cumbersome, but exact expression for R_2^{∞}.

$$R_{2,eff}^{\infty} = \frac{R_2^G + R_2^E + k_{EX}}{2} - \frac{N_{cyc}}{T_{rel}}\left(v_1 - 1 + v_3\right) - \frac{1}{T_{rel}}\ln\left(\frac{1}{2}\left(1 + e^{-2N_{cyc}v_3}\right)\left(1 + \tanh\left(N_{cyc}v_3\right)\left(\frac{v_2 + 4\tau_{cp}k_{EX}P_E}{v_3}\right)\right)\right) \quad (82)$$

By first defining $\Delta R_2^{kex} = \Delta R_2 / k_{EX}$ and:

$$T = \sqrt{2(P_G - P_E)\Delta R_2^{k_{ex}} + (\Delta R_2^{k_{ex}})^2 + 1} \quad (83)$$

Some algebra reveals that:

$$\begin{aligned} v_2^{\infty} &= 2k_{EX}\tau_{cp}\left(P_G - P_E + \Delta R_2^{k_{ex}}\right) \\ v_3^{\infty} &= 2k_{EX}\tau_{cp}T \end{aligned} \quad (84)$$

Which when substituted into equation (82) gives an exact expression for R_2^{infinity}:

$$R_{2,eff}^{\infty} = \frac{R_2^G + R_2^E + k_{EX}\left(1 - T\right)}{2} - \frac{1}{T_{rel}}\ln\left(\frac{1}{2T}\left(1 + e^{-T_{rel}k_{EX}T}\right)\left(T + \tanh\left(\frac{T_{rel}k_{EX}T}{2}\right)\left(1 + \frac{\Delta R_2}{k_{EX}}\right)\right)\right) \quad (85)$$

The logarithmic term can be neglected only when $T_{rel}k_{EX}T >> 1$. An interesting limit is if the square of T is completed from equation (83):

$$T = \left(1 + \Delta R_2^{kex}\right)\sqrt{1 - \frac{4p_E\Delta R_2^{kex}}{\left(1 + \Delta R_2^{kex}\right)^2}} \quad (86)$$

And expand in the limit $1 >> 4P_E\Delta R_2 k_{EX}\left(k_{EX} + \Delta R_2\right)^{-2}$, true if either P_E is small or if either $k_{EX} >> \Delta R_2$ or $k_{EX} << \Delta R_2$, keeping only leading terms:

$$T \approx \left(1 + \Delta R_2^{kex}\right)\left(1 - \frac{2p_E\Delta R_2^{kex}}{\left(1 + \Delta R_2^{kex}\right)^2}\right) = 1 + \Delta R_2^{kex} - \frac{2p_E\Delta R_2^{kex}}{1 + \Delta R_2^{kex}} \quad (87)$$

Substituting this into (85), in the limit where $T_{rel}k_{EX}T >> 1$ leads to equation (54)

Supplementary Section 7. Correction for exchange during indirect evolution periods

If effects of chemical shift during signal detection are neglected then equation (8) can be used to calculate $R_{2,eff}$. The description of the experiment can be improved by taking into account effects of exchange during the indirect detection period, following the lead of Hansen et al [41]. If the CPMG

element is followed immediately by indirect evolution of time t_1, then as the two states will continue to exchange during acquisition, the observed signal will be given by:

$$I(t) = O(t_1)M(\tau_{cp})\begin{pmatrix} P_G \\ P_E \end{pmatrix} \tag{88}$$

By contrast, if the indirect dimension precedes the CPMG element then:

$$I(t) = M(\tau_{cp})O(t_1)\begin{pmatrix} P_G \\ P_E \end{pmatrix} \tag{89}$$

In the former case:

$$\frac{I_G(T_{rel})}{I_G(0)} = \frac{\left(M(0,0)P_G + M(0,1)P_E\right)\left(O_E - k_{ge}\right) + \left(M(1,0)P_G + M(1,1)P_E\right)\left(O_G - k_{ge}\right)}{P_G\left(O_G - k_{eg}\right) + P_E\left(O_E - k_{ge}\right)} \tag{90}$$

And in the latter case:

$$\frac{I_G(T_{rel})}{I_G(0)} = \frac{\left(M(0,0) + M(1,0)\right)\left(P_G O_E + P_E k_{eg}\right) + \left(M(0,1) + M(1,1)\right)\left(P_E O_G + P_G k_{ge}\right)}{\left(P_G O_E + P_E k_{eg}\right) + \left(P_E O_G + P_G k_{ge}\right)} \tag{91}$$

The two are not equivalent. Using these expressions in conjunction with *M* (equation (46)), and equation (1) will lead to an improved theoretical description of the experiment.

Supplementary Section 8. Comparison to existing algorithms

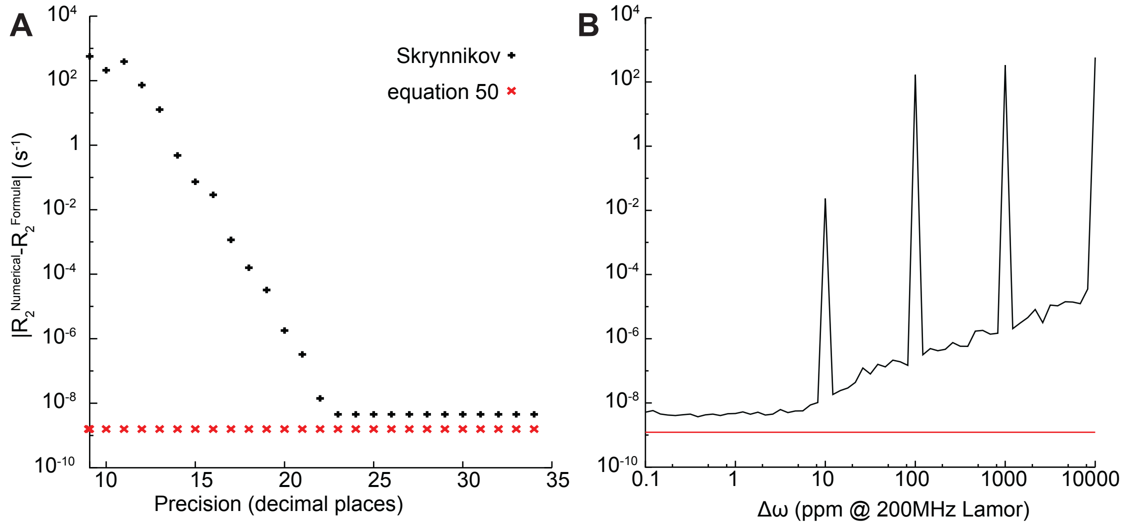

A computer derived algorithm that amounts to an exact solution for $R_{2,eff}$ has been described previously [37]. An implementation of this algorithm calculated at double floating point precision in MATLAB was found to result in erroneous values of $R_{2,eff}$ under certain combinations of parameters. This was revealed by calculating the error function as shown in the above figure, and working out its maximum value over an extensive array of the parameters: Δω (0.1 to 10,000 ppm at a Lamor frequency of 200 MHz), k_{ex}(1 to 10,000 s^{-1}), p_E (0.001 to 40%). At double floating point precision, corresponding to an accuracy of approximately 9 decimal places, the maximum error was significant, on the order of 100 s^{-1} (A, black). When the precision of the calculation was increased to 23 decimal places and beyond, the algorithm was found to give a result in agreement with both the numerical treatment, and equation 50 (A, red). To determine for which regions of parameter space the errors were incurred when using double floating-point precision, the errors were projected on $\Delta\omega$ (B). Numerical instabilities were found to affect results for $\Delta\omega$ values above approximately 10 ppm at 200MHz Lamor frequency (corresponding to 2.1ppm at 950 MHz) in a relatively unpredictable fashion. To compare the two approaches further, an implementation in C was produced containing both this algorithm[37] computed at long double precision (18 decimal places, leading to a maximum error on the order of 10^{-4} s^{-1}, A) and equation 50 evaluated at double floating point precision. The implementation of equation 50 resulted in a more compact algorithm (23 lines versus 78 lines) and was found to run approximately 12x faster on an i7 processor (code available on request). Algorithms based on equation 50 (appendix 1) can be considered exact when evaluated at double floating point precision, as used in common languages such as MATLAB and python.
